# Measures and Metrics for Feasibility of Proof-of-Concept Studies With Human Immunodeficiency Virus Rapid Point-of-Care Technologies: The Evidence and the Framework

**DOI:** 10.1097/POC.0000000000000147

**Published:** 2017-11-14

**Authors:** Nitika Pant Pai, Tiago Chiavegatti, Rohit Vijh, Nicolaos Karatzas, Jana Daher, Megan Smallwood, Tom Wong, Nora Engel

**Affiliations:** From the *Department of Medicine, McGill University; †Division of Clinical Epidemiology, McGill University Health Centre, Montreal, Quebec; ‡Dalla Lana School of Public Health, University of Toronto, Canada; and §Department of Health, Ethics and Society, Research School for Public Health and Primary Care, Maastricht University, Maastricht, The Netherlands.

**Keywords:** metrics, measures, framework, feasibility, point-of-care

## Abstract

**Objective:**

Pilot (feasibility) studies form a vast majority of diagnostic studies with point-of-care technologies but often lack use of clear measures/metrics and a consistent framework for reporting and evaluation. To fill this gap, we systematically reviewed data to (*a*) catalog feasibility measures/metrics and (*b*) propose a framework.

**Methods:**

For the period January 2000 to March 2014, 2 reviewers searched 4 databases (MEDLINE, EMBASE, CINAHL, Scopus), retrieved 1441 citations, and abstracted data from 81 studies. We observed 2 major categories of measures, that is, implementation centered and patient centered, and 4 subcategories of measures, that is, feasibility, acceptability, preference, and patient experience. We defined and delineated metrics and measures for a feasibility framework. We documented impact measures for a comparison.

**Findings:**

We observed heterogeneity in reporting of metrics as well as misclassification and misuse of metrics within measures. Although we observed poorly defined measures and metrics for feasibility, preference, and patient experience, in contrast, acceptability measure was the best defined. For example, within feasibility, metrics such as consent, completion, new infection, linkage rates, and turnaround times were misclassified and reported. Similarly, patient experience was variously reported as test convenience, comfort, pain, and/or satisfaction. In contrast, within impact measures, all the metrics were well documented, thus serving as a good baseline comparator. With our framework, we classified, delineated, and defined quantitative measures and metrics for feasibility.

**Conclusions:**

Our framework, with its defined measures/metrics, could reduce misclassification and improve the overall quality of reporting for monitoring and evaluation of rapid point-of-care technology strategies and their context-driven optimization.

## Abstract 2

Supplemental digital content is available in the text.

Recently, in the context of implementation research with point-of-care technologies/rapid diagnostic tests (POCTs/RDTs) for human immunodeficiency virus (HIV), a discussion on clear reporting of measures and metrics beyond accuracy and impact has intensified. Against this backdrop, 2 broad categories of measures have been observed in the deployment of POCT-based strategies: (1) implementation research–centered outcomes (IROs), feasibility, and impact measures and (2) patient-centered outcomes (PCOs) (ie, preference, acceptability, patient experience measures).^1–3^ Although impact and accuracy measures remain clearly defined in literature, in contrast, a concurrent lack of clarity in documentation and reporting of measures/metrics for feasibility persists.^4^ Although feasibility studies form the bulk of diagnostic literature, their measures/metrics merit a scrutiny. Although new and well-defined measures/metrics such as test efficacy rate continue to be proposed, they are rarely deployed.^4–8^ Existing checklists have focused on reporting only on test accuracy (ie, Standards for Reporting of Diagnostic Accuracy),^9^ study quality (Grading of Recommendations, Assessment, Development and Evaluation),^10^ or reporting of biases (Quality Assessment of Diagnostic Accuracy Studies). We observed a persistent lack of clarity on feasibility measures/metrics and patient-reported outcomes (acceptability, preference, patient experience).^11^ Inconsistencies in definitions for measures/metrics also compound confusion, and the absence of a reporting framework often results in misuse and misclassification, consequently impacting study and metric reporting quality.^12^ Feasibility studies are often chosen for transition to scale, and a clear reporting framework for metrics is pertinent. Clarity in metrics will aid objectives, power, and sample size estimations. In addition to the wide variety of benchmarks used to document feasibility, inconsistencies in definitions and creative reporting, either related to the processes or effect of strategies, have led to improper use of definitions. Moreover, a lack of clarity on which metric to use in which context persists in the extant literature, either in relation to research and design of studies or in the implementation of programs.^13^ Taken together, these inconsistencies and the inbuilt heterogeneity therein impact the overall quality of research, its quantification, and, furthermore, policy recommendations that emerge from scientific evidence. This reveals a lack of basic understanding of the optimal usage for metrics, especially in studies that evaluate POCT-based diagnostics and linked treatment.

Proof-of-concept studies (pilot/feasibility) are particularly relevant in diagnostics. Pilots provide a holistic assessment of performance of a program/device/initiative before a controlled trial or quasirandomized impact assessment–based scale-up study can be planned or conducted. Pilots are very popular, in part because it is difficult to mount trials with time/resource constraints and unclear impacts on clinical decisions and patient wellness decisions. A vast majority of pilot studies explore feasibility and patient-centered outcomes. Patient-centered outcomes are also in evolution.

With the recent shift in research on diagnostics taking center stage in developing settings for improving the quality of care, and in parallel in developed settings with companion, molecular diagnostics for personalized medicine, and emergent threat of antimicrobial resistance, these measures/metrics needed to be revisited. In this context, we felt a need to synthesize evidence and harmonize the reporting of outcome measures/metrics. Furthermore, to respond to the need, we proposed a reporting framework to inform funding, policy decisions, and guideline development for POCT pilots. In an era where real-time diagnosis at the point of clinical care is rapidly becoming mainstream, the time to clarify such measures and metrics, beyond accuracy and impact, is long overdue. With this in mind, our objective was to call for standardized reporting of measures/metrics used in HIV POCTs/RDTs and propose a reporting framework.

## METHODS

Our specific aims were the following:

1. To underline the heterogeneity in reporting, measuring, and defining measures and metrics related to feasibility and patient-reported/centered outcomes, and
2. To develop an improved framework of reporting and documentation with a goal to develop the overall quality of reporting for pilot studies (Table 1 refers to our framework).

**TABLE 1:**
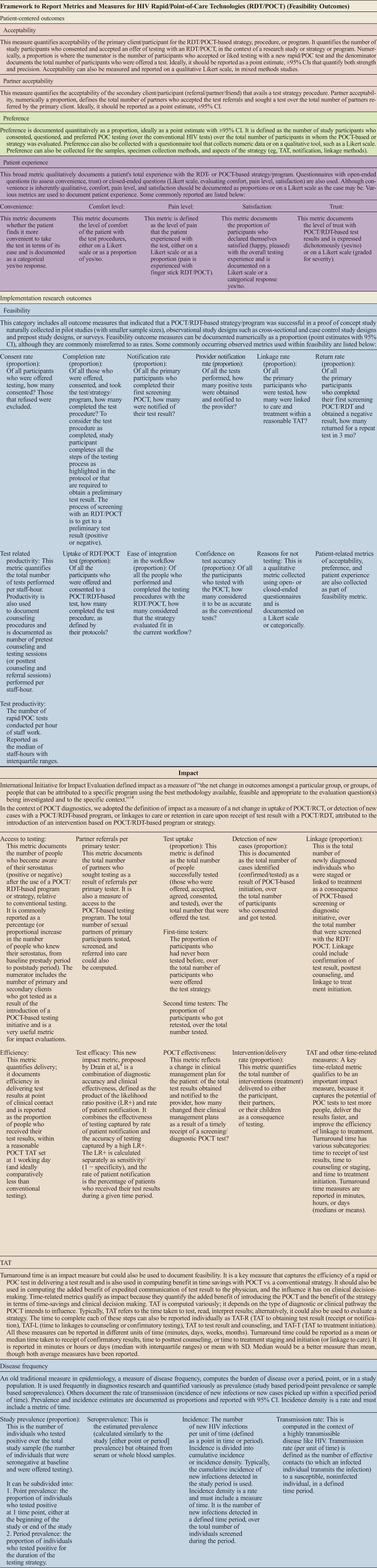
Framework for Reporting of Measures and Metrics

Recently, we classified outcomes for syphilis POCTs beyond accuracy. We organized outcomes into 2 broad categories: (*a*) IROs, feasibility, and prevalence and (*b*) PCOs, that is, acceptability, preference, patient experience, etc.^6^ Impact measures have been reported for a comparison. In this systematic review, we revisit the framework and reporting of metrics and measures for HIV POCTs/RDTs. We collated and synthesized all available evidence and aligned it as per a framework.

### Search Methodology

We systematically searched published literature on rapid tests and POCTs for HIV from January 1, 2000 to March 6, 2014. We searched for data in 4 electronic databases: MEDLINE, EMBASE, CINAHL, and Scopus.

Our search string was the following: HIV [MeSH] OR Acquired Immunodeficiency Syndrome [MeSH], OR “HIV Antigens” [tiab], OR “HIV Antibodies” [tiab]) AND (“rapid test” [tiab] OR “point-of-care” [tiab] OR “test” [tiab]) AND (“acceptability” [tiab] OR “preference” [tiab] OR “cost” [tiab] OR “feasibility” [tiab] OR “concordance” [tiab], OR “prevalence” [tiab] OR “impact” [tiab] OR “field performance” [tiab]).

**FIGURE 1:**
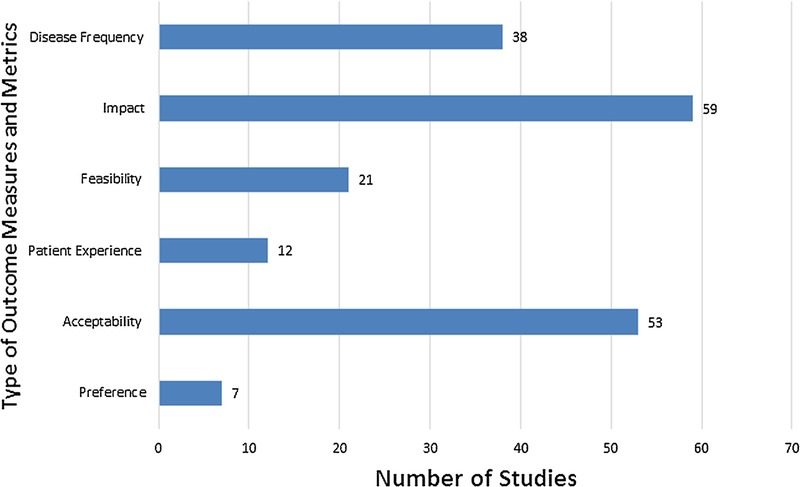
Distribution of included studies by measures. This figure can be viewed online in color at www.poctjournal.com.

We followed the Cochrane methodology for systematic reviews. Our search strategy aimed to review all studies that documented any measure or metric related to implementation of HIV testing strategies using rapid and POCT tests. Two reviewers (T.C. and R.V.) independently screened and reviewed the full text of the articles and abstracted data. Criteria for study inclusion were determined by discussion among 2 primary reviewers, and, in cases of reviewer discordance, a third reviewer was consulted (N.P.P.). Figure 1 illustrates our study selection process.

Studies were considered eligible if they satisfied all of the following criteria:

1. Documented the use of HIV point-of-care or rapid tests;
2. Evaluated at least 1 implementation research– or patient-centered outcome;
3. Were conducted in humans or in human samples;
4. Were written in English, French, Spanish, or Portuguese.

### Exclusion Criteria

Editorials, news reports, reviews, modeling studies, and studies that only evaluated laboratory tests/surveys on risk behavior were excluded. Data were abstracted from studies published in English (n = 78) and in French, Spanish, or Portuguese (n = 3) using a standardized data abstraction form and reporting framework created for this review. We collected metrics for each measure, evaluated them against our framework (refer to Table 1), proposed working definitions,^6^ and subclassified metrics. We included mixed methods studies^15–27^ but excluded those analyzing costs (economic outcomes) for a separate review.

## RESULTS

Please refer to Tables 1A–E of included studies (see Tables 1A–E, Supplemental Digital Content, http://links.lww.com/POC/A14).

A total of 81 studies met our inclusion criteria (refer to Fig. 1). These studies evaluated either IRO and/or PCO. Within IRO, 59 studies accounted for impact measures that were documented for a comparison. Of the remaining studies, 38 reported disease frequency measures and 21 documented feasibility measures. Among PCO, 53 studies reported on acceptability, 12 reported on patient experience, and 7 on preference measures (we included impact measures to serve as a reference for a comparison with feasibility measures).

### Acceptability

In our framework (refer to Table 1), we defined acceptability as a proportion: the number of primary clients who consented and accepted to be tested with a POCT over the total number of participants in the study, strategy, or program.

Of the 53 studies reporting on acceptability measure, 81% (n = 43/53) documented it well and counted only acceptability of tests as a metric, but 15% of studies (n = 8/53)^21,28–34^ misclassified it; they counted refusal to test as acceptability. The other 2 studies^35,36^ combined within acceptability several processes like consent, testing, and study procedures.^37^ We classified flow of participants throughout a study and documented these metrics with greater clarity. Confusion on what defines acceptability prevailed in 15% studies. Furthermore, 4 studies incorrectly referred to acceptability as a rate (a misnomer; not a proportion).^23,38–40^ Other studies were creative in the use of acceptability, with use of metrics such as partner testing^41^ or the number of visits needed to test.^42^ Regarding precision, 81% (47/53) of studies documented these metrics as a proportion, but only 3 (6%) reported the precision with 95% confidence intervals (CIs).^21,24,43^

### Preference

As per our framework (refer to Table 1), we defined preference as the proportion of study participants who preferred the POCT or rapid test strategy/program over the conventional HIV test/strategy/program. Only 1 study accurately described preference in line with our framework.^40^

Within preference, various metrics and comparators were reported by studies. Of 7 studies, 5 (71%) reported preference for type of testing strategy (ie, POCT only vs conventional). The remaining 2 studies reported on another metric, as in preference for the number of POCT tests performed,^44^ preference for test site,^40^ or preference for the type of specimen used, instead of preference for the POCT strategy itself. Other preference metrics were preference for the time to receive the POC test results or preference for the receipt of test results.^45–47^ Furthermore, 2 studies misclassified preference: either reporting it as uptake, which is an impact measure,^48^ or reporting preference as the “quality of test experience.”^49^ Five studies explored reasons to prefer POCT,^15,40,46,50,51^ either qualitatively, on a Likert scale,^40,46^ or quantitatively,^15,50,51^ with an odds ratio (with 95% CIs).^46^

### Patient Experience

Patient experience is largely a qualitative outcome/measure but was also expressed quantitatively in many of the included studies. As per our framework, of 81 studies, 12 (15%) reported patient experience with various metrics including satisfaction, access, convenience, and level of comfort. The Likert scale was used in only 1 study to evaluate the overall satisfaction with POCT^52^; patient experience was also documented using preference for test sample in 4 studies. As for 2 other studies (3%), ease of test execution,^41,53^ patient's level of comfort,^53^ and access and convenience of POCT were reported.^41^

### Feasibility

Our framework defines feasibility as a category encompassing outcome measures that indicate how successful a POCT/RDT-based strategy or program is, in a context in which the strategy/program/intervention was evaluated in a population group and in a small proof-of-concept study (refer to Table 1).

Following our definition, 21 (26%) of 81 studies reported on feasibility; however, the definitions of feasibility varied across studies. Two studies (2/21, 10%) concluded that the test or strategy was feasible without any data nor metrics to support this claim.^39,54^ Various metrics were used to report and define the feasibility outcome, including among others consent rate, completion rate,^42^ uptake,^55^ and offer rate (3/21, 14%).^42,56,57^ For example, in 1 study, an offer rate was defined as the proportion of those who were offered the test over the total eligible patients.^56,57^ In another study, offer rate was defined as the proportion of patient visits during which testing was offered^42^ or as “missed opportunities” in the third study.^57^

Heterogeneity in reporting persisted in definitions and documentation, impairing clarity. For example, completion rate (of test procedure) was reported in 4 studies (19% of the 21 observed)^33,42,56,58^ but defined in only 2.^56,58^ Whereas in 1 study it was reported as a percentage of women tested during labor,^33^ in another it was reported as test completion rate per patient visit. Numerators and denominators changed adding to heterogeneity.^42^ Likewise, the return rate was documented in 3 studies and reported inconsistently, either as (1) the proportion of individuals tested who returned for posttest counseling,^59^ (2) the proportion of individuals who successfully retested after having deferred testing,^60^ or (3) the proportion of individuals who received a repeat test.^30^ The linkage metric was also documented inconsistently depending on the type of posttest linkage initiated (eg, referral, care/treatment, counseling) and reported as the proportion of referrals to HIV care^16^ or the number (not proportion) of infected women who received treatment.^61^ Besides quantitative reporting, the qualitative documentation of measures was also impaired. Measures such as ease of testing (as in procedure),^16,50,58^ workflow integration^19,29,52,62,63^ (38%), the impressions of participants,^16^ perception of patients,^52^ perceptions of performance^58^ (2/81, 3%), and the ease of test execution^41,53^ were reported. These measures also need to be defined.

Other feasibility metrics:

### Turnaround Time

Turnaround time (TAT) measures capture the efficiency of the test in delivering a result and can be computed in several ways depending on the type of diagnostic or clinical pathway that the POCT aims to influence. Turnaround time typically refers to how long it takes to test, read, and interpret the results, but the time to complete each of these steps can also be reported separately. Alternatively, TAT may refer to how long it takes to complete a specific step of the clinical pathway, such as the time to receive a confirmatory test result, time to receive posttest counseling, time to treatment initiation, or time to staging (or linkage to care). Across studies, TAT was defined in terms of availability of test result and reported in 3 studies.^16,49,64^ In 1 study, TAT was documented qualitatively.^16^ Only 1 study proposed a clear definition for TAT. Three different metrics were related to TAT: (1) proportion of tests results available within 1 hour, (2) median test duration, and (3) time between sample collection.^49^

### Productivity

Productivity appeared in 2 studies and was defined differently. In 1 study, it was reported as the total number of tests carried out per staff-hour,^65^ and the other defined productivity as the mean number of visits per patient (reported as mean ± SD).^66^

### Trust

On this measure, study participants were asked whether they would choose a POCT in the future and whether they trusted their test results; the results were either reported as proportions or using Likert scores. Two studies documented patient confidence on the accuracy of POCT.^50,52^

### Test Volume

Test volume refers to the volume of tests performed in a defined time period. For this measure, 1 study documented the change in the annual demand for HIV tests and the change in ordering tests.^38^ Other studies documented the change in the number of patients seeking rapid testing.^28,30^

### Rapid Test Awareness

One study reported on the increase in awareness of rapid tests, before and after the introduction of the tests.^28^

### Impact (as a Comparator of Measure/Metrics Within it)

Impact definitions have been clarified by the International Initiative for Impact Evaluation (3ie). Impact has been defined by 3ie as “the net change in outcomes amongst a particular group, or groups of people that can be attributed to a specific program using the best methodology available, feasible and appropriate to the evaluation question(s) being investigated and to the specific context.”^14^ This definition is very broad and encompasses a range of contexts, settings, programs, and interventions. We documented them as a comparator to demonstrate the contrast in reporting of feasibility metrics and measures.

Of 81 studies, 59 (73%) reported on a total of 163 impact metrics, with some studies often reporting 2 metrics. We classified these metrics into the following categories: uptake, detection of new cases, first time testers, receipt rate (proportion), linkage rate (proportion), intervention delivery rate (proportion), partner notification rate (proportion), referral rate (proportion), and TAT. Of these, detection of new cases was the most common metric (72/163, 44%), followed by first time testers (21/163, 13%), test result receipt rate (19/163, 12%), linkage rate (16/163, 10%), and test delivery rate (15/163, 9%). Uptake, TAT, partner notification, and referrals accounted for only 12% (20/163) of impact measures. Only 3 studies reported metrics perfectly in line with our framework.^25,39,67^

In terms of break up, metrics were separately reported as follows, and in some studies, these metrics were mixed up or creatively reported. (*a*) Increase in uptake: Uptake of testing was documented by 2 studies, that is, Anaya et al^68^ and Herbert et al,^63^ but reportedly misclassified as *testing rate*. Metsch et al^69^ reported on the likelihood (as adjusted risk ratio with CIs) of completing POCT strategy as uptake, whereas a third documented the proportion of participants tested as uptake.^70^ (*b*) Receipt of test results: 14 studies reported the receipt of test results as a proportion, 2 as a rate,^25,28^ and 3 documented the likelihood of receipt as an odds^70^ or risk ratio^25,71^ with 95% CIs. (*c*) New case detection: A total of 41 studies documented detection of new cases (proportion) often without CIs. (*d*) Rate of delivery of linked intervention: Rate was reported in 10 studies, documented in detail in 6,^72^ and reported variously as either a cumulative probability,^21^ sometimes accurately as a rate^73^ or as the number of cases where test results were not received in time with POCT;^49^ as the number of patients whose treatment changed because of a positive POCT result,^38^ or a decrease in unnecessary postexposure prophylaxis among health care workers with POCT.^26^ (*e*) First time testers: One of the best-defined metrics, reported in 18 (30%) of 59 studies as the proportion of those who were being tested for the first time often without CIs;^72^ one study reported it as a number alone,^40^ whereas another reported it as missed opportunities.^74^ (*f*) Linkage (proportion): Linkage was defined inconsistently, either as a proportion of patients who adhered to their first medical appointment or of those who completed follow-up.^38,51,75^ Only 1 study reported linkages with CIs,^43^ and another as a “high proportion of failure to return for confirmatory testing.”^23^ (*g*) Test efficiency: Test efficiency was documented by 2 studies as the proportion of actionable test results^49^ or those test results that were “resolved” at a screening visit.^22^ (*h*) Turnaround time: The TAT was defined inconsistently, either defined as the time taken to test,^49,72,76^ the total time to referral to an intervention,^72^ or the time between sample collection and test result.^49^ Turnaround time was reported as a median or a range.^49,72^ (*i*) Partner notification: Partner notification or referral rates (proportions) were documented in only 4 studies. Notification was reported either as the number^19^ or as the proportion^72^ of participants who disclosed their serostatus with their partners or as the proportion of patients who would recommend an HIV self-test to others.^48^ Partner referral was also documented qualitatively.^41^ (*j*) Mortality (testing rate): Ashby et al^77^ and van Rooyen et al^55^ documented the number of deaths as part of roll out of testing strategy. Of 45, only 7 studies (16%) reported 95% CIs.

### Measures of Disease Frequency (as a Comparator)

Precise definitions for measures of disease frequency are defined in many epidemiology textbooks. Prevalence was the most commonly reported measure, but only 10 (26%) of 38 studies reported it with 95% CIs;^78^ the remaining 28 studies were unclear, with 1 study reporting it as a relative risk.^50^ Period prevalence was defined accurately by 6 studies.^79^ A study confused the concepts of prevalence and incidence, reporting it as a new measure, “prevalence/rate of new incidence.”^67^ Incidence, on the other hand, was well defined.^65^ Transmission rate was not clearly reported.^80^

## DISCUSSION

Using our proposed framework for feasibility, with clear standardized definitions (refer to Table 1), we attempted to reclassify and reevaluate metrics for feasibility, and patient-centered outcomes of preference, acceptability, and patient experience, that were reported with HIV point-of-care and rapid technologies. Across all studies, we observed heterogeneity and variability in reporting of various outcomes, inconsistent definitions, and documentation, with resultant misclassification of outcomes and measures.

Although feasibility, preference, and patient experience were the most frequently confused measures, acceptability was the best defined among them. Impact as a comparator was best defined. We attributed clarity in reporting impact to clear definitions outlined by the 3ie initiative.^14^

Another key finding was a lack of clarity on which metric to use, when, how, and in which context to use it; confusion prevailed, and careless numeric reporting of point estimates from feasibility studies without CIs was observed. Creative definitions and erroneous documentation generated confusion as to what was attempted, documented, and reported. Despite the reporting of a well-defined new impact measure called the test efficacy, the metric was not used at all by any study.^4^ This explains the disconnect in the application of clear metrics in diagnostics.

Oftentimes, qualitative research on patient experience with the POCT strategy provides a meaningful assessment of the utility of the strategy, compared with quantitative research with unclear metrics and measures.^81,82^ In this regard, a lack of clarity on the application of qualitative research metrics within mixed designs was also observed.

**FIGURE 2:**
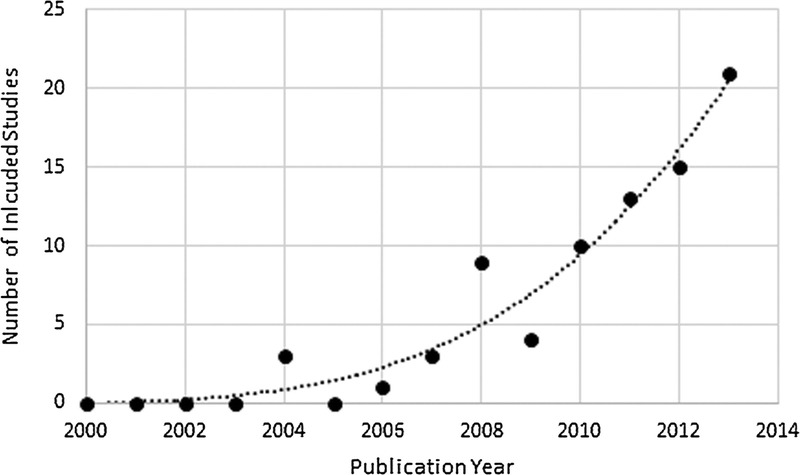
Number of included studies by publication year.

Incidentally, a time trend in reporting of outcomes beyond accuracy has been observed. Although the number of studies increased over time (refer to Fig. 2), the quality of reporting of measures/metrics remained unchanged. Although trends changed, test device evaluations were replaced by evaluations of test strategies/programs over time. Although our feasibility framework is aimed to improve clarity in reporting, the proposed measures/metrics will require a greater integration within observational and pilot trial designs. This framework could be adapted to other POCT initiatives targeted to other key sexually transmitted and blood borne infections (eg, hepatitis C virus, hepatitis B virus, syphilis, human papillomavirus, herpes simplex virus, chlamydia/gonorrhea) in the near future.

We do hope that new POCT devices will incorporate electronic documentation of measures/metrics with a digital data log in real time that automatically computes, plots, and displays key measures/metrics. This process will aid implementation and encourage donor agencies to monitor and document the impact of their interventions. This process will also reduce the extent of misclassification and further minimize errors in reporting of simple measures like proportions and TAT.

### Strengths and Limitations of Review

A comprehensive search and use of a strong methodology were our strengths. Publication bias cannot be ruled out.

### Implications for Research and Policy

This feasibility framework is aimed for pilot studies. It will be of interest to various stakeholders (ie, researchers, health care professionals, policy makers, laboratory professionals, funders, donors, front line health care professionals, and community-based organizations) that are involved in implementation, monitoring, and evaluation of POCT initiatives for HIV and related coinfections.

## CONCLUSIONS

With this framework, we hope to improve the quality of collection, documentation, reporting, and classification of feasibility outcomes needed to evaluate HIV POCT/RDT-based programs and strategies. Clearly defined measures, and ideally, the use of standardized metrics, will facilitate a better comparison of different strategies, evaluations, and their context-driven optimization. Our findings will find resonance in the daily work needed for global implementation of HIV POCT/RDT policies, for both clinical/implementation research and global health practice.

## Supplementary Material

SUPPLEMENTARY MATERIAL
