# Supplementary material for: Measures and Metrics for Feasibility of Proof-of-Concept Studies With Human Immunodeficiency Virus Rapid Point-of-Care Technologies: The Evidence and the Framework
Source: Point Care. 2017 Nov 14;16(4):141–50. doi: 10.1097/POC.0000000000000147 (PMC5737458; doi:10.1097/POC.0000000000000147)
Supplement: SUPPLEMENTARY MATERIAL [file poc-16-141-s001.docx]

**Measures and metrics for feasibility of proof-of-concept studies with HIV rapid point-of-care technologies: the evidence and the framework.**

**Authors:**

Nitika Pant Pai MD, MPH, PhD^1,2^, Tiago Chiavegatti PhD^2^, Rohit Vijh BSc^2^,

Nicolaos Karatzas BSc^2^, Jana Daher MSc (PH)^2^, Megan Smallwood BSc, MSc^2^

Tom Wong MD, MPH, CCFP, FRCPC^3^, Nora Engel PhD^4^

**Affiliations:**

^1^Department of Medicine, McGill University, Montreal, Quebec, Canada

^2^Division of Clinical Epidemiology, McGill University Health Centre, Montreal, Quebec, Canada

^3^Dalla Lana School of Public Health, University of Toronto, Canada.

^4^Department of Health, Ethics & Society, Research School for Public Health and Primary Care, Maastricht University, Maastricht, The Netherlands

**Corresponding Author:**

Nitika Pant Pai, M.D., M.P.H., Ph.D., Assistant Professor, Division of Clinical Epidemiology, Department of Medicine McGill University and Health Centre, 5252 boul de Maisonneuve, Montreal, Quebec, Canada H4A 3S5.

Tel: 514-934-1934 x44729/ Fax: 514-934-8293/ Email: [nitika.pai@mcgill.ca](mailto:nitika.pai@mcgill.ca)

**Conflicts of interest:** None.

**Funding:**

Gates Foundation operating grant OPP1061487; CIHR HIB-131558

FRSQ salary Award Junior 1 (2015-2018) **Abstract**

**Objective:** Pilot (feasibility) studies form a vast majority of diagnostic studies with point-of-care technologies (POCT), but often lack use of clear measures/metrics and a consistent framework for reporting and evaluation. To fill this gap, we systematically reviewed data to: a) catalogue feasibility measures/metrics, and b) propose a framework.

**Methods:** For the period January 2000 to March 2014, two reviewers searched four databases (MEDLINE, EMBASE, CINAHL, Scopus), retrieved 1441 citations, and abstracted data from 81 studies. We observed two major categories of measures: implementation centered or patient centered and 4 subcategories of measures: feasibility, acceptability, preference, and patient experience. We defined and delineated metrics and measures for a feasibility framework. We documented impact measures for a comparison.

**Findings:**

We observed heterogeneity in reporting of metrics as well as misclassification and misuse of metrics within measures. While we observed poorly defined measures and metrics for feasibility, preference and patient experience, in contrast, acceptability measure was the best defined. For example, within feasibility, metrics such as consent, completion, new infection, linkage rates, and turnaround times, were misclassified and reported. Similarly, patient experience was variously reported as test convenience, comfort, pain and/or satisfaction. In contrast, within impact measures, all the metrics were well documented, thus serving as a good baseline comparator. With our framework, we classified, delineated and defined quantitative measures and metrics for feasibility.

**Conclusion:** Our framework, with its defined measures/metrics, could reduce misclassification and improve the overall quality of reporting for monitoring and evaluation of rapid POCT strategies and their context-driven optimization.

**Keywords:** Metrics; measures; framework; feasibility; point-of-care.

**Introduction**

Recently, in the context of implementation research with point of care technologies/rapid diagnostic tests (POCT/RDT) for HIV, a discussion on clear reporting of measures and metrics beyond accuracy and impact has intensified. Against this backdrop, two broad categories of measures have been observed in the deployment of POCT based strategies: 1. Implementation research centered outcomes (IRO), feasibility and impact measures, and 2. Patient centered outcomes (PCO) (i.e., preference, acceptability, patient experience measures).^1-3^ While impact and accuracy measures remain clearly defined in literature, in contrast, a concurrent lack of clarity in documentation and reporting of measures/metrics for feasibility persists.^4^ Although feasibility studies form the bulk of diagnostic literature, their measures/metrics merit a scrutiny. While new and well-defined measures/metrics such as test efficacy rate continue to be proposed, they are rarely deployed.^4-8^ Existing checklists have focused on reporting only on test accuracy (i.e., STARD),^9^ study quality (GRADE),^10^ or reporting of biases (QUADAS). We observed a persistent lack of clarity on feasibility measures/metrics and patient reported outcomes (acceptability, preference, patient experience).^11^ Inconsistencies in definitions for measures/metrics also compound confusion, and the absence of a reporting framework often results in misuse and misclassification, consequently impacting study and metric reporting quality.^12^ Feasibility studies are often chosen for transition to scale and a clear reporting framework for metrics is pertinent. Clarity in metrics will aid objectives, power and sample size estimations. In addition to the wide variety of benchmarks used to document feasibility, inconsistencies in definitions and creative reporting, either related to the processes or effect of strategies, have led to improper use of definitions. Moreover, a lack of clarity on which metric to use in which context persists in the extant literature; either in relation to research and design of studies or in the implementation of programs.^13^ Taken together, these inconsistencies and the inbuilt heterogeneity therein, impact the overall quality of research, its quantification, and furthermore, policy recommendations that emerge from scientific evidence. This reveals a lack of basic understanding of the optimal usage for metrics, especially in studies that evaluate POCT based diagnostics and linked treatment.

Proof-of-concept studies (pilot/feasibility) are particularly relevant in diagnostics. Pilots provide a holistic assessment of performance of a program/device/initiative before a controlled trial or quasi randomized impact assessment based scale up study can be planned or conducted. Pilots are very popular, in part because it is difficult to mount trials with time/resource constraints and unclear impacts on clinical decisions and patient wellness decisions. A vast majority of pilot studies explore feasibility and patient centered outcomes. Patient centered outcomes are also in evolution.

With the recent shift in research on diagnostics taking center stage in developing settings for improving the quality of care, and in parallel in developed settings with companion, molecular diagnostics for personalized medicine and emergent threat of antimicrobial resistance, these measures/metrics needed to be revisited. In this context, we felt a need to synthesize evidence and harmonize the reporting of outcome measures/metrics. Furthermore, to respond to the need, we proposed a reporting framework to inform funding, policy decisions and guideline development for POCT pilots. In an era where real-time diagnosis at the point of clinical care is rapidly becoming mainstream, the time to clarify such measures and metrics, beyond accuracy and impact, is long overdue. With this in mind, our objective was to call for standardized reporting of measures/metrics used in HIV POCTs/RDTs, and propose a reporting framework.

**Methods**

Our specific aims were:

1. To underline the heterogeneity in reporting, measuring, and defining measures and metrics related to feasibility and patient reported/centered outcomes, and
2. To develop an improved framework of reporting and documentation with a goal to develop the overall quality of reporting for pilot studies (Table 1 refers to our framework).

Recently, we classified outcomes for syphilis POCTs beyond accuracy. We organized outcomes into two broad categories: a) implementation research centered outcomes (IRO), feasibility, prevalence, and b) patient-centered outcomes (PCO): acceptability, preference, patient experience, etc.^6^ Impact measures have been reported for a comparison. In this systematic review, we revisit the framework and reporting of metrics and measures for HIV POCTs/RDTs. We collated and synthesized all available evidence and aligned it as per a framework.

**Search methodology:**

We systematically searched published literature on rapid tests and POCTs for HIV from January 1st, 2000 to March 6th, 2014. We searched for data in four electronic databases: MEDLINE, EMBASE, CINAHL and Scopus.

Our search string: HIV [MeSH] OR Acquired Immunodeficiency Syndrome [MeSH], OR ‘HIV Antigens’ [tiab], OR ‘HIV Antibodies’ [tiab]) AND (‘rapid test’ [tiab] OR ‘point-of-care’ [tiab] OR ‘test’ [tiab]) AND (‘acceptability’ [tiab] OR ‘preference’ [tiab] OR ‘cost’ [tiab] OR ‘feasibility’ [tiab] OR ‘concordance’ [tiab], OR ‘prevalence’ [tiab] OR ‘impact’ [tiab] OR 'field performance‘ [tiab]).

We followed the Cochrane methodology for systematic reviews. Our search strategy aimed to review all studies that documented any measure or metric related to implementation of HIV testing strategies using rapid and POCT tests. Two reviewers (TC and RV) independently screened and reviewed the full text of the articles and abstracted data. Criteria for study inclusion were determined by discussion among two primary reviewers, and, in cases of reviewer discordance, a third reviewer was consulted (NPP). Figure 1 illustrates our study selection process.

Studies were considered eligible if they satisfied all of the following criteria:

1. Documented the use of HIV point-of-care or rapid tests;
2. Evaluated at least one implementation research or patient-centered outcome;
3. Were conducted in humans or in human samples;
4. Were written in English, French, Spanish or Portuguese.

**Exclusion criteria:**

Editorials, news reports, reviews, modeling studies and studies that only evaluated laboratory tests/surveys on risk behavior were excluded. Data were abstracted from studies published in English (n=78) and in French, Spanish, or Portuguese (n=3) using a standardized data abstraction form and reporting framework created for this review. We collected metrics for each measure, evaluated them against our framework (refer to Table 1), proposed working definitions,^6^ and sub-classified metrics. We included mixed methods studies,^14-26^ but excluded those analyzing costs (economic outcomes) for a separate review.

**Results**

Please refer to Table1a-e of included studies (see Tables, Supplemental Digital Content, http://links.lww.com/POC/A14).

81 studies met our inclusion criteria (refer to Figure 1). These studies evaluated either IRO and/or PCO. Within IRO, 59 studies accounted for impact measures that were documented for a comparison. Of the remaining studies, 38 reported disease frequency measures and 21 documented feasibility measures. Among PCO, 53 studies reported on acceptability, 12 reported on patient experience and 7 on preference measures (we included impact measures to serve as a reference for a comparison with feasibility measures).

**Acceptability:** In our framework (refer to Table 1), we defined acceptability as a proportion: the number of primary clients who consented and accepted to be tested with a POCT over the total number of participants in the study, strategy or program.

Of the 53 studies reporting on acceptability measure, 81% (n= 43/53) documented it well and counted only acceptability of tests as a metric, but 15% of studies (n=8/53)^20, 27-33^ misclassified it; they counted refusal to test as acceptability. The other two studies^34,35^ combined within acceptability several processes like consent, testing and study procedures.^36^ We classified flow of participants throughout a study, and documented these metrics with greater clarity. Confusion on what defines acceptability prevailed in 15% studies. Furthermore, four studies incorrectly referred to acceptability as a rate (a misnomer; not a proportion).^22,37-39^ Other studies were creative in the use of acceptability, with use of metrics such as partner testing^40^ or the number of visits needed to test.^41^ Regarding precision, 81% of studies (47/53) documented these metrics as a proportion, but only 3 (6 %) reported the precision with 95% confidence intervals.^20, 23,42^

**Preference:** As per our framework (refer to Table 1), we defined preference as the proportion of study participants who preferred the POCT or rapid test strategy/program over the conventional HIV test/strategy/program. Only one study accurately described preference in line with our framework.^39^

Within preference, various metrics and comparators were reported by studies. Of seven studies, five (71 %) reported preference for type of testing strategy (i.e., POCT only vs. conventional). The remaining two studies reported on another metric, as in preference for the number of POCT tests performed,^43^ preference for test site,^39^ or preference for the type of specimen used, instead of preference for the POCT strategy itself. Other preference metrics were preference for the time to receive the POC test results, or preference for the receipt of test results.^44-46^ Furthermore, two studies misclassified preference: either reporting it as uptake, which is an impact measure,^47^ or reporting preference as the “quality of test experience”.^48^ Five studies explored reasons to prefer POCT;^14,39,45,49,50^ either qualitatively, on a Likert scale,^39,45^ or quantitatively,^14,49,50^ with an odds ratio (with 95% confidence intervals).^45^

**Patient experience:** Patient experience is largely a qualitative outcome/measure, but was also expressed quantitatively in many of the included studies. As per our framework, of 81 studies, 12 (15%) reported patient experience with various metrics including satisfaction, access, convenience, and level of comfort. The Likert scale was used in only one study to evaluate the overall satisfaction with POCT;^51^ patient experience was also documented using preference for test sample in four studies. As for two other studies (3 %), ease of test execution,^40,52^ patient’s level of comfort,^52^ as well as access and convenience of POCT were reported.^40^

**Feasibility:** Our framework defines feasibility as a category encompassing outcome measures that indicate how successful a POCT/RDT based strategy or program is, in a context in which the strategy/program/intervention was evaluated in a population group and in a small proof-of-concept study (refer to Table 1).

Following our definition, 21 of 81 studies (26%) reported on feasibility, however the definitions of feasibility varied across studies. Two studies (2/21; 10%) concluded that the test or strategy was feasible without any data nor metrics to support this claim.^38,53^ Various metrics were used to report and define the feasibility outcome, including among others: consent rate, completion rate,^41^ uptake,^54^ and offer rate (3/21; 14 %).^41,55,56^ For example, in one study, an offer rate was defined as the proportion of those who were offered the test over the total eligible patients.^55,56^ In another study, offer rate was defined as the proportion of patient visits during which testing was offered,^41^ or as “missed opportunities” in the third study.^56^

Heterogeneity in reporting persisted in definitions and documentation, impairing clarity. For example, completion rate (of test procedure) was reported in 4 studies (19 % of the 21 observed),^32,41,55,57^ but defined in only two.^55,57^ While in one study it was reported as a percentage of women tested during labor,^32^ in another it was reported as test completion rate per patient visit. Numerators and denominators changed adding to heterogeneity.^41^ Likewise, the return rate was documented in three studies and reported inconsistently; either as 1) the proportion of individuals tested who returned for post-test counseling, ^58^ 2) the proportion of individuals who successfully re-tested after having deferred testing,^59^ or 3) the proportion of individuals who received a repeat test.^29^ The linkage metric was also documented inconsistently depending on the type of post-test linkage initiated (e.g., referral, care/treatment, counseling) and reported as the proportion of referrals to HIV care^15^ or the number (not proportion) of infected women that received treatment.^60^ Besides quantitative reporting, the qualitative documentation of measures was also impaired. Measures such as ease of testing (as in procedure),^15,49,57^ workflow integration^18,28,51,61,62^ (38 %), the impressions of participants,^15^ perception of patients,^51^ perceptions of performance^57^ (2/81; 3%) and the ease of test execution^40,52^ were reported. These measures also need to be defined.

Other Feasibility metrics:

**Turnaround time (TAT)**: TAT measures capture the efficiency of the test in delivering a result, and can be computed in several ways depending on the type of diagnostic or clinical pathway that the POCT aims to influence. TAT typically refers to how long it takes to test, read, and interpret the results, but the time to complete each of these steps can also be reported separately. Alternatively, TAT may refer to how long it takes to complete a specific step of the clinical pathway, such as the time to receive a confirmatory test result, time to receive post-test counseling, time to treatment initiation, or time to staging (or linkage to care). Across studies, TAT was defined in terms of availability of test result and reported in three studies.^15,48, 63^  In one study, TAT was documented qualitatively.^15^ Only one study proposed a clear definition for TAT. Three different metrics were related to TAT: 1) proportion of tests results available within one hour; 2) median test duration; and 3) time between sample collection.^48^

**Productivity:** Productivity appeared in two studies and was defined differently. In one study, it was reported as the total number of tests carried out per staff-hour,^64^ and the other defined productivity as the mean number of visits per patient (reported as mean ± SD).^65^

**Trust:** On this measure, study participants were asked whether they would choose a POCT in the future and whether they trusted their test results; the results were either reported as proportions or using Likert scores. Two studies documented patient confidence on the accuracy of POCT.^49,51^

**Test Volume:** The volume of tests performed in a defined time period. For this measure, one study documented the change in the annual demand for HIV tests and the change in ordering tests.^37^ Other studies documented the change in the number of patients seeking rapid testing.^27, 29^

**Rapid Test Awareness:** One study reported on the increase in awareness of rapid tests, before and after the introduction of the tests.^27^

**Impact (as a comparator of measure/metrics within it)**

Impact definitions have been clarified by the International Initiative for Impact Evaluation (3ie). Impact has been defined by 3ie as “the net change in outcomes amongst a particular group, or groups of people that can be attributed to a specific program using the best methodology available, feasible and appropriate to the evaluation question(s) being investigated and to the specific context.”^80^ This definition is very broad and encompasses a range of contexts, settings, programs and interventions. We documented them as a comparator to demonstrate the contrast in reporting of feasibility metrics and measures.

Of 81 studies, 59 (73%) reported on a total of 163 impact metrics, with some studies often reporting two metrics. We classified these metrics into the following categories: uptake, detection of new cases, first time testers, receipt rate (proportion), linkage rate (proportion), intervention delivery rate (proportion), partner notification rate (proportion), referral rate (proportion), and turnaround time (TAT). Of these, detection of new cases was the most common metric (72/163; 44 %), followed by first time testers (21/163; 13 %), test result receipt rate (19/163; 12 %), linkage rate (16/163; 10 %), and test delivery rate (15/163; 9 %). Uptake, TAT, partner notification, and referrals accounted for only 12 % (20/163) of impact measures. Only three studies reported metrics perfectly in line with our framework.^24,38,66^

In terms of break up, metrics were separately reported as follows and in some studies these metrics were mixed up or creatively reported. **a. Increase in uptake:** Uptake of testing was documented by two studies: Anaya et al.^67^ and Herbert et al.^62^ but reportedly misclassified as *testing rate*. Metsch et al. reported on the likelihood (as adjusted risk ratio with confidence intervals) of completing POCT strategy as uptake,^68^ while a third documented the proportion of participants tested as uptake.^69^ **b. Receipt of test results:** 14 studies reported the receipt of test results as a proportion, two as a rate,^24,27^ and three documented the likelihood of receipt as an odds^69^ or risk ratio^24,70^ with 95% confidence intervals. c**. New case detection:** 41 studies documented detection of new cases (proportion) often without confidence intervals. **d. Rate of delivery of linked intervention:** Rate was reported in ten studies, documented in detail in six,^71^ and reported variously as either a cumulative probability^20^, sometimes accurately as a rate^72^ or as the number of cases where test results were not received in time with POCT;^48^ as the number of patients whose treatment changed because of a positive POCT result,^37^ or a decrease in unnecessary post-exposure prophylaxis (PEP) among health care workers with POCT.^25^ **h. First time testers:** One of the best defined metrics, reported in 18 out of 59 studies (30 %) as the proportion of those who were being tested for the first time often without confidence intervals;^71^ one study reported it as a number alone,^39^ while another reported it as missed opportunities.^73^ **i. Linkage (proportion)**: Linkage was defined inconsistently, either as a proportion of patients who adhered to their first medical appointment, or of those who completed follow up.^37,50,74^ Only one study reported linkages with confidence intervals,^42^ and another as a “high proportion of failure to return for confirmatory testing”.^22^  **j. Test efficiency:** Test efficiency was documented by two studies as the proportion of actionable test results^48^ or those test results that were “resolved” at a screening visit.^21^ **k. Turnaround time (TAT):** TAT was defined inconsistently; either defined as the time taken to test,^48,71,75^ the total time to referral to an intervention,^71^ or the time between sample collection and test result.^48^ TAT was reported as a median or a range.^48,71^ **l. Partner notification:** Partner notification or referral rates (proportions) were documented in only four studies. Notification was reported either as the number^18^ or as the proportion^71^ of participants who disclosed their sero-status with their partners, or as the proportion of patients who would recommend an HIV self-test to others.^47^ Partner referral was also documented qualitatively.^40^ **m. Mortality (testing rate):** Ashby et al. and van Rooyen et al. documented the number of deaths as part of roll out of testing strategy.^54,76^ Of 45, only seven studies (16%) reported 95 % confidence intervals.

**Measures of Disease Frequency (as a comparator)**

Precise definitions for measures of disease frequency are defined in many epidemiology textbooks. Prevalence was the most commonly reported measure, but only 10 (26 %) of 38 studies reported it with 95 % confidence intervals;^77^ the remaining 28 studies were unclear, with one study reporting it as a relative risk.^49^ Period prevalence was defined accurately by 6 studies.^78^ A study confused the concepts of prevalence and incidence, reporting it as a new measure, “prevalence/rate of new incidence”.^66^ Incidence, on the other hand, was well defined.^64^ Transmission rate was not clearly reported.^79^

**Discussion**

Using our proposed framework for feasibility, with clear standardized definitions (refer to Table 1), we attempted to re-classify and re-evaluate metrics for feasibility, and patient centered outcomes of preference, acceptability and patient experience, that were reported with HIV point of care and rapid technologies. Across all studies, we observed heterogeneity and variability in reporting of various outcomes, inconsistent definitions and documentation, with resultant misclassification of outcomes and measures.

While feasibility, preference, and patient experience were the most frequently confused measures, acceptability was the best defined amongst them. Impact as a comparator was best defined. We attributed clarity in reporting impact to clear definitions outlined by the 3ie initiative.^80^

Another key finding was a lack of clarity on which metric to use, when, how, and in which context to use it; confusion prevailed, and careless numeric reporting of point estimates from feasibility studies without confidence intervals was observed. Creative definitions and erroneous documentation generated confusion as to what was attempted, documented and reported. Despite the reporting of a well-defined new impact measure called the test-efficacy, the metric was not used at all by any study.^4^ This explains the disconnect in the application of clear metrics in diagnostics.

Oftentimes, qualitative research on patient experience with the POCT strategy provides a meaningful assessment of the utility of the strategy, compared to quantitative research with unclear metrics and measures.^81,82^ In this regard, a lack of clarity on the application of qualitative research metrics within mixed designs was also observed.

Incidentally, a time trend in reporting of outcomes beyond accuracy has been observed. Although the number of studies increased over time (refer to Figure 2), the quality of reporting of measures/metrics remained unchanged. While trends changed, test device evaluations were replaced by evaluations of test strategies/programs over time. Although our feasibility framework is aimed to improve clarity in reporting, the proposed measures/metrics will require a greater integration within observational and pilot trial designs. This framework could be adapted to other POCT initiatives targeted to other key sexually transmitted and blood borne infections (e.g., HCV, HBV, Syphilis, HPV, HSV, CT/GC) in the near future.

We do hope that new POCT devices will incorporate electronic documentation of measures/metrics with a digital data log in real time that automatically computes, plots and displays key measures/metrics. This process will aid implementation and encourage donor agencies to monitor and document the impact of their interventions. This process will also reduce the extent of misclassification and further minimize errors in reporting of simple measures like proportions and TAT.

*Strengths and limitations of review:*

A comprehensive search and use of a strong methodology were our strengths*.* Publication bias cannot be ruled out.

*Implications for Research and policy:*

This feasibility framework is aimed for pilot studies. It will be of interest to various stakeholders (i.e. researchers, health care professionals, policy makers, laboratory professionals, funders, donors, front line health care professionals and community based organizations) that are involved in implementation, monitoring and evaluation of POCT initiatives for HIV and related co-infections.

**Conclusion**

With this framework, we hope to improve the quality of collection, documentation, reporting, and classification of feasibility outcomes needed to evaluate HIV POCT/RDT based programs and strategies. Clearly defined measures, and ideally, the use of standardized metrics, will facilitate a better comparison of different strategies, evaluations and their context-driven optimization. Our findings will find resonance in the daily work needed for global implementation of HIV POCT/RDT policies, for both clinical/implementation research and global health practice.

**References**

1. St-Louis P. Status of point-of-care testing: promise, realities, and possibilities. *Clin Biochem*. 2000;33(6):427-440.

2. Pai NP, Sollis K, Peeling RW. Rapid hepatitis C tests: better than the gold standard? *Exp Rev Mol Diagn*. 2013;13(3):221-223.

3. Banoo S, Bell D, Bossuyt P, et al. Evaluation of diagnostic tests for infectious diseases: general principles. *Nature Rev Microbiol.* 2008;6(11 Suppl):S16-26.

4. Drain PK, Hyle EP, Noubary F, et al. Diagnostic point-of-care tests in resource-limited settings. *Lancet Infect Dis.* 2014;14(3):239-49

5. Fontela PS, Pai NP, Schiller I, Dendukuri N, Ramsay A, Pai M. Quality and reporting of diagnostic accuracy studies in TB, HIV and malaria: evaluation using QUADAS and STARD standards. *PLoS One.* 2009;4(11):e7753

6. Jafari Y, Johri M, Joseph L, Vadnais C, Pant Pai N. Poor Reporting of Outcomes Beyond Accuracy in Point-of-Care Tests for Syphilis: A Call for a Framework. *AIDS Res Treat*. 2014;2014:465932.

7. White H. Theory-based impact evaluation: principles and practice. Journal of development effectiveness. 2009;1(3):271-84

8. Whiting P, Rutjes AW, Reitsma JB, Bossuyt PM, Kleijnen J. The development of QUADAS: a tool for the quality assessment of studies of diagnostic accuracy included in systematic reviews. *BMC Med Res Meth.* 2003;3(1):25

9. Bossuyt PM, Reitsma JB, Bruns DE, et al. The STARD statement for reporting studies of diagnostic accuracy: explanation and elaboration. *Ann Intern Med.* 2003;138(1):W1-12

10. Guyatt GH, Oxman AD, Vist GE, et al. GRADE: an emerging consensus on rating quality of evidence and strength of recommendations. *BMJ* *Clin Res ed.* 2008;336(7650):924-926.

11. Whiting PF, Rutjes AW, Westwood ME, et al. QUADAS-2: a revised tool for the quality assessment of diagnostic accuracy studies. *Ann Intern Med.* 2011;155(8):529-36

12. Hänscheid T, Rebelo M, Grobusch MP. Point-of-care tests: where is the point? *Lancet Infect* *Dis.* 2014;14(10):922.

13. Pai NP, Balram B, Shivkumar S, et al. Head-to-head comparison of accuracy of a rapid point-of-care HIV test with oral versus whole-blood specimens: a systematic review and meta-analysis. *Lancet Infect Dis.* 2012;12(5):373-80

14. Pai NP, Barick R, Tulsky JP, et al. Impact of round-the-clock, rapid oral fluid HIV testing of women in labor in rural India. *PLoS Med.* 2008; 5(5): e92.

15 Becker ML, Thompson LH, Pindera C, et al. Feasibility and success of HIV point-of-care testing in an emergency department in an urban Canadian setting. *Can J Infect Dis Med.* 2013; 24(1): 27-31.

16. Burns F, Edwards SG, Woods J, et al. Acceptability, feasibility and costs of universal offer of rapid point of care testing for HIV in an acute admissions unit: results of the RAPID project. *HIV Med*. 2013;14 Suppl 3:10-14.

17. Carballo-Dieguez A, Frasca T, Balan I, Ibitoye M, Dolezal C. Use of a rapid HIV home test prevents HIV exposure in a high risk sample of men who have sex with men. *AIDS Behav.* 2012;16(7):1753-1760.

18. Conners EE, Hagedorn HJ, Butler JN, et al. Evaluating the implementation of nurse-initiated HIV rapid testing in three Veterans Health Administration substance use disorder clinics. *Int J STD AIDS.* 2012; 23(11): 799-805.

19. Mayhood MK, Afwamba IA, Odhiambo CO, et al. Validation, performance under field conditions, and cost-effectiveness of Capillus HIV-1/HIV-2 and determine HIV-1/2 rapid human immunodeficiency virus antibody assays using sequential and parallel testing algorithms in Tanzania. *J Clin Microbiol.* 2008;46(12):3946-3951.

20. Melo M, Varella I, Castro A, et al. HIV voluntary counseling and testing of couples during maternal labor and delivery: The TRIPAI couples study. *Sex Transm Dis.* 2013; 40(9): 704-9.

21. Morin SF, Khumalo-Sakutukwa G, Charlebois ED, et al. Removing barriers to knowing HIstatus: same-day mobile HIV testing in Zimbabwe. *J Acquir Immune Defic Syndr.* 2006; 41(2): 218-24.

22. Mungrue K, Sahadool S, Evans R, et al. Assessing the HIV rapid test in the fight against the HIV/AIDS epidemic in Trinidad. *HIV/AIDS (Auckland, NZ).*  2013; 5: 191-8.

23. Noble H, Wright G, Young E. HIV point of care testing in the emergency department. *HIV Med.* 2012; 13: 61.

24. Thomas R, Machouf N, Trottier B, et al. A new approach to encourage HIV testing in high-risk populations at the clinique l'actuel. *Sex Transm Infect.* 2011; 87: A201.

25. Hoyos J, de la Fuente L, Fernandez S, et al. [Street outreach rapid HIV testing in university settings: a priority strategy?]. *Gac Sanit.* 2012; 26(2): 131-7.

26. Hoyos J, Fernandez-Balbuena S, de la Fuente L, et al. Never tested for HIV in Latin-American migrants and Spaniards: prevalence and perceived barriers. *J Int AIDS Soc.* 2013;16:18560.

27. Kania D, Fao P, Valéa D, et al. Low prevalence rate of indeterminate serological human immunodeficiency virus results among pregnant women from Burkina Faso, West Africa. *J Clin Microbiol.* 2010; 48(4): 1333-6.

28. Levin M, Mathema H, Stinson K, Jennings K. Acceptability, feasibility and impact of routine screening to detect undiagnosed HIV infection in 17 - 24-month-old children in the western sub-district of Cape Town. *S Afr Med J.* 2012; 102(4): 245-8.

29. Macgowan R, Margolis A, Richardson-Moore A, et al. Voluntary rapid human immunodeficiency virus (HIV) testing in jails. *Sex Transm Dis.* 2009; 36(2 Suppl): S9-13.

30. Manavi K, Williams G, Newton R. The uptake of HIV and syphilis testing in a nurse-delivered service during Gay Pride events. *Int J STD AIDS.* 2012; 23(12): 887-9.

31. Martin EG, Salaru G, Paul SM, Cadoff EM. Use of a rapid HIV testing algorithm to improve linkage to care. *J Clin Virol* 2011; 52 Suppl 1: S11-5.

32. Mathe MK, Rigo J, Sontag D, Gerard C. Prevalence of HIV infection among pregnant women. A study in rural Africa. [French] Prevalence de l'infection par le VIH chez les femmes enceintes. Etude en milieu rural africain. *Rev Epidemiol Sante Publique.* 2008; 56(6): 407-13.

33. Melvin AJ, Alarcon J, Velasquez C, et al. Rapid HIV type 1 testing of women presenting in late pregnancy with unknown HIV status in Lima, Peru. *AIDS Res Hum Retroviruses.* 2004; 20(10): 1046-52.

34. Jabbari H, Aghamollaie S, Esmaeeli Djavid G, et al. Frequency of HIV Infection among Sailors in South of Iran by Rapid HIV Test. *AIDS Res Treat.* 2011; 2011: 612475.

35. Menacho I, Sequeira E, Muns M, et al. Comparison of two HIV testing strategies in primary care centres: Indicator-condition-guided testing vs. testing of those with non-indicator conditions. *HIV Medicine.* 2013; 14(SUPPL.3): 33-7.

36. Mkwanazi NB, Patel D, Newell ML, et al. Rapid testing may not improve uptake of HIV testing and same day results in a rural South African community: a cohort study of 12,000 women. *PLoS One.* 2008; 3(10): e3501.

37. Mullins TLK, Braverman PK, Dorn LD, Kollar LM, Kahn JA. Adolescent preferences for human immunodeficiency virus testing methods and impact of rapid tests on receipt of results. *J Adolescent Health.* 2010; 46(2): 162-8.

38. Ndondoki C, Brou H, Timite-Konan M, et al. Universal HIV Screening at Postnatal Points of Care: Which Public Health Approach for Early Infant Diagnosis in Cote d'Ivoire? *PLoS One.* 2013; 8(8).

39. Nelson AK, Caldas A, Sebastian JL, et al. Community-based rapid oral human immunodeficiency virus testing for tuberculosis patients in Lima, Peru. *Am J Trop Med Hyg*. 2012; 87(3): 399-406.

40. Gennotte AF, Semaille P, Ellis C, et al. Feasibility and acceptability of HIV screening through the use of rapid tests by general practitioners in a Brussels area with a substantial African community. *HIV Med.* 2013; 14(SUPPL.3): 57-60.

41. Newbould C, Monrose C, Dodge J, et al. Don't forget the children - Ongoing experience of a paediatric HIV unit using point-of-care tests in children born to HIV-positive parents - How far have we come? *HIV Med.* 2010; 11: 67-8.

42. Ouladlahsen A, Bensghir R, Karkouri M, et al. [Benefit of the rapid test determine HIV1/2 in the clinical diagnosis of HIV infection in Ibn Rochd hospital of Casablanca, Morocco]. *Rev Epidemiol Sante Publique.* 2012; 60(4): 333-8.

43. Marsh KA, Reynolds GL, Rogala BE, Fisher DG, Napper LE. Who chooses a rapid test for HIV in Los Angeles County, California? *Eval Health Prof.* 2010; 33(2): 177-96.

44. Jabbari H, Sharifi AH, SeyedAlinaghi S, et al. Assessing the prevalence of HIV among Afghan immigrants in Iran through rapid HIV testing in the field. *Acta Med Iran.* 2011; 49(7): 478-9.

45. Sattin RW, Wilde JA, Freeman AE, Miller KM, Dias JK. Rapid HIV testing in a southeastern emergency department serving a semiurban-semirural adolescent and adult population. *Ann Emerg Med.* 2011; 58(1 Suppl 1): S60-4.

46. Scognamiglio P, Chiaradia G, Sciarrone MR, et al. Final results of an outreach program of HIV rapid testing among marginalized people living in Rome, Italy. *Infection* 2011; 39: S24.

47. Ramachandran R, Chandrasekaran V, Muniyandi M, Jaggarajamma K, Bagchi A, Sahu S. Prevalence and Risk Factors of HIV Infection among Clients Attending ICTCs in Six Districts of Tamilnadu, South India. *AIDS Res Treat.* 2011; 2011: 650321.

48. Guenter D, Greer J, Barbara A, Robinson G, Roberts J, Browne G. Rapid point-of-care HIV testing in community-based anonymous testing program: a valuable alternative to conventional testing. *AIDS Patient Care STDS.* 2008; 22(3): 195-204.

49. Robbins CL, Zapata L, Kissin DM, et al. Multicity HIV seroprevalence in street youth, Ukraine. *Int J STD AIDS.* 2010; 21(7): 489-96.

50. Ruutel K, Ustina V, Parker RD. Piloting HIV rapid testing in community-based settings in Estonia. *Scand J Public Health.* 2012; 40(7): 629-33.

51. Garrard N, Peck J, Ruf M, Lockyer S. Opt-out HIV testing pilot in termination of pregnancy services - 11-month service evaluation. *HIV Med.* 2010; 11: 69.

52. Tepper NK, Farr SL, Danner SP, et al. Rapid human immunodeficiency virus testing in obstetric outpatient settings: the MIRIAD study. *Am J Obstet Gynecol.* 2009; 201(1): 31 e1-6.

53. Theron GB, Shapiro DE, Van Dyke R, et al. Rapid intrapartum or postpartum HIV testing at a midwife obstetric unit and a district hospital in South Africa. *Int J Gynaecol. Obstet* 2011; 113(1): 44-9.

54. van Rooyen H, Barnabas RV, Baeten JM, et al. High HIV testing uptake and linkage to care in a novel program of home-based HIV counseling and testing with facilitated referral in KwaZulu-Natal, South Africa. *J Acqur Immune Defic Syndr.* 2013; 64(1): e1-8.

55. Veloso VG, Bastos FI, Portela MC, et al. HIV rapid testing as a key strategy for prevention of mother-to-child transmission in Brazil. *Rev Saude Publica.* 2010; 44(5): 803-11.

56. Viani RM, Araneta MRG, Spector SA. Parallel rapid HIV testing in pregnant women at Tijuana General Hospital, Baja California, Mexico. *AIDS Res Hum Retroviruses.* 2013; 29(3): 429-34.

57. White DA, Scribner AN, Schulden JD, Branson BM, Heffelfinger JD. Results of a rapid HIV screening and diagnostic testing program in an urban emergency department. *Ann Emerg Med.* 2009; 54(1): 56-64.

58. Young PW, Mahomed M, Horth RZ, Shiraishi RW, Jani IV. Routine data from prevention of mother-to-child transmission (PMTCT) HIV testing not yet ready for HIV surveillance in Mozambique: a retrospective analysis of matched test results. *BMC Infect Dis.* 2013; 13: 96.

59. Choko AT, Desmond N, Webb EL, et al. The uptake and accuracy of oral kits for HIV self-testing in high HIV prevalence setting: a cross-sectional feasibility study in Blantyre, Malawi. *PLoS Med.* 2011; 8(10): e1001102.

60. Castel AD, Magnus M, Peterson J, et al. Implementing a novel citywide rapid HIV testing campaign in Washington, D.C.: findings and lessons learned. *Public Health Rep.* 2012; 127(4): 422-31.

61. Gaydos CA, Solis M, Hsieh YH, Jett-Goheen M, Nour S, Rothman RE. Use of tablet-based kiosks in the emergency department to guide patient HIV self-testing with a point-of-care oral fluid test. *Int J STD AIDS.* 2013; 24(9): 716-21.

62. Herbert R, Ashraf AN, Yates TA, et al. Nurse-delivered universal point-of-care testing for HIV in an open-access returning traveller clinic. *HIV Med.* 2012; 13(8): 499-504.

63. Nobrega I, Dantas P, Rocha P, et al. Syphilis and HIV-1 among parturient women in Salvador, Brazil: Low prevalence of syphilis and high rate of loss to follow-up in HIV-infected women. *Braz J Infect Dis.* 2013; 17(2): 184-93.

64. Russell TV, Do AN, Setik E, et al. Sexual risk behaviors for HIV/AIDS in Chuuk State, Micronesia: the case for HIV prevention in vulnerable remote populations. *PLoS One.* 2007; 2(12): e1283.

65. Parisi MR, Soldini L, Vidoni G, et al. Cross-sectional study of community serostatus to highlight undiagnosed HIV infections with oral fluid HIV-1/2 rapid test in non-conventional settings. *New Microbiol.* 2013; 36(2): 121-32.

66. Seewald R, Bruce RD, Elam R, et al. Effectiveness and feasibility study of routine HIV rapid testing in an urban methadone maintenance treatment program. *Am J Drug Alcohol Ab.* 2013; 39(4): 247-51.

67. Anaya H, Feld J, Hoang T, Knapp H, Asch S. Implementing an HIV rapid testing intervention for homeless veterans in shelter settings within Los Angeles county. *J Int Ass Physicians in AIDS Care.* 2010; 9 (1): 47.

68. Metsch LR, Feaster DJ, Gooden L, et al. Implementing rapid HIV testing with or without risk-reduction counseling in drug treatment centers: results of a randomized trial. *Am J Public Health.* 2012; 102(6): 1160-7.

69. Keller S, Jones J, Erbelding E. Choice of Rapid HIV testing and entrance into care in Baltimore City sexually transmitted infections clinics. *AIDS Patient Care STDS.* 2011; 25(4): 237-43.

70. Benzaken A, Pinto VM, Carvalho CH, Peeling R. Increasing access to hiv and syphilis screening in remote areas using rapid tests. *Sex Transm Infect.* 2011; 87: A2.

71. Ekouevi DK, Kariyiare BG, Coffie PA, et al. Feasibility and acceptability of rapid HIV screening in a labour ward in Togo. *J Int AIDS Soc.* 2013; 15(2).

72. Ganesan A, Thatchinamoorthy G, Saramini S. Feasibility of testing antibodies to HIV from filter paper using HIV rapid test kits. *J Int AIDS Soc.* 2010; 13.

73. Jerene D, Endale A, Lindtjorn B. Acceptability of HIV counselling and testing among tuberculosis patients in south Ethiopia. *BMC Int Health Hum Rights.* 2007; 7: 4.

74. Mikolasova G, Bonnach C, Monte CTW, et al. Relative low number of new hiv cases detected in rural district Bunda in Northwest Tanzania. *Am J Trop Med Hyg.* 2013; 1): 234.

75. Mwembo-Tambwe ANK, Kalenga MK, Donnen P, et al. HIV testing among women in delivery rooms in Lubumbashi, DR Congo: A catch-up strategy for prevention of mother-to-child transmission. [French] Depistage du VIH en salle de travail a Lubumbashi, Republique democratique du Congo. Une strategie de rattrapage dans le cadre de la prevention de la transmission de la mere a l'enfant. *Revue d'Epidemiologie et de Sante Publique.* 2013; 61(1): 21-7.

76. Ashby J, Braithewaite B, Walsh J, Gnani S, Fidler S, Cooke G. HIV testing uptake and acceptability in an inner city polyclinic. *AIDS Care.* 2012; 24(7): 905-9.

77. Qvist T, Cowan SA, Graugaard C, Helleberg M. High linkage to care in a community-based rapid HIV testing and counseling project among men who have sex with men in copenhagen. *Sex Transm Dis.* 2014; 41(3): 209-14.

78. Anaya HD, Hoang T, Golden JF, et al. Improving HIV screening and receipt of results by nurse-initiated streamlined counseling and rapid testing. *J Gen Intern Med.* 2008; 23(6): 800-7.

79. Beckwith CG, Liu T, Bazerman LB, et al. HIV risk behavior before and after HIV counseling and testing in jail: a pilot study. *J Acquir Immune Defic Syndr.* 2010; 53(4): 485-90.

80. International Initiative for Impact Evaluation (3ie). 3ie Principles for Impact Evaluation2012-04-20, 2008. <http://www.3ieimpact.org/media/filer_public/2012/04/20/principles-for-impact-evaluation.pdf> (accessed 2014-02-15).

81. Hernandez B, Mateo N, Walter J, Setterholm K. Targeted bedside emergency department hiv screening does not impact length of stay. *Acad Emerg Med.* 2013; (1): S200.

82. Engel N, Davids M, Blankvoort N, Dheda K, Pant Pai N, Pai M. Making HIV testing work at the point of care in South Africa: a qualitative study of diagnostic practices. *BMC Health Serv Res.* 2017;17(1):408.

**Table and Figure Legends**

**Table 1.** Framework for reporting of measures and metrics

**Figure 1.** Distribution of included studies by measures

**Figure 2.** Number of included studies by publication year
