# Supplementary material for: Measures and Metrics for Feasibility of Proof-of-Concept Studies With Human Immunodeficiency Virus Rapid Point-of-Care Technologies: The Evidence and the Framework
Source: Point Care. 2017 Nov 14;16(4):141–50. doi: 10.1097/POC.0000000000000147 (PMC5737458; doi:10.1097/POC.0000000000000147)
Supplement: SUPPLEMENTARY MATERIAL [file poc-16-141-s003.pdf]

Table 1a: Table of included studies on acceptability

Study	Setting	Study design and population	Outcome Reported	Definition of acceptability used by the authors	Reporting
Anaya et al., 2010 [1]	9 shelters of the Los Angeles Homeless Services Authority	Randomized controlled trial with homeless veterans using the shelters	97/136 (71.3%)	Proportion of participants that accepted testing	Proportion; no interval estimation
Arfai et al., 2011[2]	Urban ED	Survey with patients above 13 years-old	406/2384 (17%)	Proportion of participants that consented to test	Proportion; no interval estimation
Ashby et al., 2010[3]	United Kingdom	Survey with patients of a polyclinic serving a highly migrant urban population	71/93 (76%)	Number of individuals that accepted to test among those that were approached	Proportion; no interval estimation
Batey et al., 2012[4]	Alabama, USA	Interviews with individuals presenting to a Level I trauma and academic medical center	186/247 (64.6%)	Proportion of participants that consented to test	Proportions; no interval estimation
Beckwith et al., 2011[5]	Rhode Island, USA	Rhode Island Department of Corrections inmates; interviews with key informants (nurse, doctors) and focus groups with correctional staff	1343/1364 (98%)	Proportion of participants that completed rapid HIV testing among those that were offered a rapid test; also defined as the proportion of participants that accepted and consented to testing, and as acceptance rate	Proportions; no interval estimation
Bowles et al., 2008[6]	Boston; Chicago; Detroit; Kansas City, Missouri; Los Angeles; San Francisco; and Washington, D.C.	Cohort of adults with unknown serostatus	60% [Not all sites systematically collected data on the number of people who declined testing (Detroit and Washington only).]	Acceptance rate, calculated by dividing the number of people that accepted testing by the total number of people approached for testing during the periods in which refusal data were collected	Proportions; no interval estimation
Bucher et al., 2007[7]	San Francisco, USA	Cohort of homeless and marginally housed adults	95% (95% CI = 88.7–94.7%) agreed to the test when testing occurred at the site of recruitment vs 70.9% (95% CI = 68.3–73.4%) when test was at a nearby site (P<0.0001). Acceptance rate was 87.2% in shelters, 72.9% in meal lines, and 70.5% in single room occupancy hotels (P<0.0001).	Acceptance rate, calculated as the proportion of individuals that agreed to rapid testing	Proportions with 95% CI
Burns et al., 2013[8]	Central acute medical admissions unit in London, England	Mixed methods study (implementation and survey) with adults (19-95 yrs. old)	153/282 (54.3%) agreed to participate in the study. 93.6% (131 of 140) agreed to a test. 97% of participants thought POCT HIV testing was both a good idea and appropriate. 90.1% liked receiving information via video.	Proportion of: participants that agreed to participate; participants that thought that the POCT was good idea or appropriate; participants that liked receiving information via video	Proportions; no interval estimation

Carballo-Diéguez et al., 2012[9]	New York City, USA	Mixed methods study (diagnostic evaluation and survey) with MSM	101/124 of partners accepted to test	Partner acceptance to home-based test	Proportions; no interval estimation
Choko et al., 2011[10]	Urban Blantyre, Malawi	Cohort of adult (>=16 yrs.) members of 60 households and 72 members of community peer groups	Self-testing options were ranked second to door-to-door standard VCT by an external provider as being most likely to successfully increase HIV testing in the community. Local distribution of self-test kits by a neighbor without having to disclose results was acceptable to 205 (94.5%) of participants.	Acceptance of strategy	Proportions; no interval estimation
Cirone et al., 2013[11]	Urban level 1 trauma center (presumably at the US)	Cohort of ED patients (18-64 yrs.); pilot study	75/80 (95%) accepted HIV rescreening with the rapid HIV test	Proportion of participants that consented to test	Proportions; no interval estimation
Crinti et al., 2009[12]	Ambulatory obstetrics and gynecology clinic, Philadelphia, PA	Survey with women (18-45 yrs.) at 32 weeks or more of gestation and a documented HIV negative test before 24 weeks of gestation	75/80 (95%) accepted HIV rescreening with the rapid HIV test; accepted test because: testing was good for the baby (76%), were encouraged by provider (40%); declined test because: did not perceive themselves at risk (n=3) , tested for HIV recently (n=2) and reported sexual abstinence since first HIV test (n=2)	Proportion of participants that accepted or that declined testing	Proportions; no interval estimation
Darling et al., 2012[13]	Red Lights District in Lausanne, Switzerland	Survey with clients of FSW (all were men)	n=112, n=127 and n=79 men were interviewed in 2009, 2010 and 2011 respectively, of which n= 30, 64, 37 were agreed to HIV testing but only n=31, 47 and 31 were actually tested (others did not follow through because they found the waiting time unacceptable)	Proportion of participants that agreed or refused to test	Number of individuals
Ekouevi et al., 2012[14]	Tokoin Teaching Hospital in Lomé (Togo)	Survey with pregnant women	Acceptance of HIV testing in the labor ward: 91.9% (n=467); among those with antenatal testing record: 90.8% (326/359); among those who tested for the first time in labor ward: 94.6% (141/149) (p=0.15)	Proportion of participants that accepted testing (acceptance was compared among women who tested for the first time in labor ward and those with records of antenatal testing)	Proportions; no interval estimation
Garrard et al., 2010[15]	United Kingdom	Implementation study in a cohort of women attending a termination of pregnancy service who were recommended HIV tests as part of	Of the 2831 women attending the service between November 2008 and September 2009, 36.9% (n=1044) had a HIV test documented	Proportion of participants that had a documented HIV test	Proportions; no interval estimation

		routine consultation			
Gaydos et al., 2013[16]	Johns Hopkins Hospital ED (Baltimore, USA)	Survey with emergency department patients (18–64 yrs. old)	473/955 (49.5%)	Proportion of participants that consented to participate (among those who were approached)	Proportions; no interval estimation
Gennotte et al., 2013[17]	10 medical centers in Belgium	Cohort of patients with an indicator condition, AIDS-defining illness, belonging to a high-prevalence group, having returned from a country with a high HIV prevalence, having had a recent pregnancy or abortion, or with other risk for HIV	12/217 (6%) refused the standard test because they were not covered by national health insurance or fear of losing anonymity; 13/217 (6%) refused rapid testing because it was too stressful or because they were not ready to receive a result immediately	Proportion of participants that declined to test	Proportions; no interval estimation
Herbert et al., 2012[18]	Hospital for Tropical Diseases, London, UK	Before-after study in a cohort of patients of the open-access emergency clinic	44.8% vs.23.6% (p<0.0001)	Acceptance of rapid test (compared to conventional testing)	Proportions; no interval estimation
Hooshyar et al., 2014[19]	Dallas, Fort Worth, and Texoma (Texas, USA)	Cohort of homeless veterans attending to an outreach event	133/910 veterans were tested	Proportion of participants that were tested	Proportions; no interval estimation
Jabbari et al., 2011[20]	Shahid Rajaie, Lengeh, and Shahid Bahonar ports (south of Iran)	Mixed methods (cohort and survey) study with sailors	400/409	Proportion of participants that consented to test of all those that were approached	Proportions; no interval estimation
Jabbari et al., 2011[21]	Lavasan (northeast of Tehran, Iran)	Surveillance study with immigrant Afghan population (11 years or older) living in Lavasan, Iran	477/491 (97.1%)	Acceptance rate	Proportions; no interval estimation
Kania et.al 2010[22]	Bobo-Dioulasso (Burkina Faso, West Africa)	Diagnostic evaluated among ARV-naïve pregnant women secreened for HIV in order to participate in the PMTCT Kesho Bora trial	44653/51983 (85.9%)	Proportion of participants that accepted to be screened for HIV	Proportions; no interval estimation
Levin et al., 2012[23]	Cape Town, South Africa	Diagnostic evaluation among caregivers and previously untested children (aged 17–24 months) attending to immunization clinics	499/567 caregivers consented.	Proportion of participants that consented to test	Proportions; no interval estimation
Macgowan et al., 2009[24]	Florida, Louisiana, New York, and Wisconsin	Implementation study in a cohort of jail inmates	422/440 (96%)	Proportion of participants with positive rapid test who accepted to take a confirmatory testing	Proportions; no interval estimation

Manavi et al., 2012[25]	Birmingham Pride event	Cohort; authors mention only consenting adults (men)	398/405 (98%)	Proportion of individuals that agreed to be tested; test uptake	Proportions; no interval estimation
Martin et al., 2011[26]	24 sites in New Jersey	Diagnostic evaluation with clients of health facilities in the rapid testing program	25/394	Proportion of preliminary positives participants that refused confirmatory WB	Proportions; no interval estimation
Mathe et al., 2008[27]	Rural hospital in North East RDC	Cohort of pregnant women undergoing counselling for HIV (part of a large study examining the risks of postnatal HIV transmission associated with different modes of infant feeding)	47/2609 (1.8%) refused to test (among those who refused to test, 1 said she was afraid of the needle, and 3 said they were against testing without giving a specific reason)	Proportion of participants that declined testing	Proportions; no interval estimation
Melo et al., 2013[28]	Public hospital in Porto Alegre, Brazil	Mixed methods (cohort + survey) study with pregnant women and their partners	1648/2888 (95.3%) women accepted enrollment and 81 (4.7%) refused; 1094 men accepted HIV rapid testing (66.4%) and 554 (33.6%) declined testing; 1648 (57%) women consented to partner testing	Enrollment; proportion of participants that accepted or that declined testing; consent to partner testing	Proportions; no interval estimation
Melvin et al., 2004[29]	Instituto Materno-Perinatal in Lima, Peru	Diagnostic evaluation with previously untested pregnant women presenting to the emergency room that were not beyond the first stage of labor	All but one of the women approached for testing consented for the investigation and completed the study protocol	Consent to test	Number of Individuals
Menacho et al., 2013[30]	Primary care centers in Barcelona, Spain	Mixed methods (cohort + survey) study with adult patients attending to primary care clinics (with an indicator condition (herpes zoster, seborrheic eczema, mononucleosis syndrome and leucopenia/thrombocytopenia) vs. without	Indicator condition cohort: 85/89 (94%); control cohort: 313/344 (90%)	Proportion of participants that accepted testing	Proportions; no interval estimation
Mkwanazi et al. 2008[31]	8 rural clinics in KwaZulu-Natal (South Africa)	Cohort of pregnant women undergoing counselling for HIV (part of a large study examining the risks of postnatal HIV transmission associated with different modes of infant feeding)	4810/6444 (74.6%)	Proportion of participants that accepted testing	Proportions; no interval estimation
Mullins et al., 2010[32]	Cincinnati, Ohio	Survey with adolescents were recruited from an urban hospital-based adolescent primary care clinic	200/399 (50%)	Proportion of participants that agreed to test	Proportions; no interval estimation

Mungrue et al., 2012[33]	Queen's Park Counselling Centre and Clinic in Trinidad	Mixed methods (diagnostic evaluation + survey) with all persons seeking HIV testing at the facility	247/297 (83.2%)	Consent to pre-test counseling	Proportions; no interval estimation
Ndondoki et al., 2013[34]	Abidjan, Côte d'Ivoire	Survey with children aged 6–26 weeks attending community clinics and their parents/caregivers	1817 mothers (60.9%; 95%CI: 59.1%–62.6%) accepted their own postpartum HIV test; 35/46 fathers presenting with infant accepted to test (76.1%; 95%CI: 63.8%–88.4%)	Proportion of participants that accepted testing; proportion of infants with formal parental consent among those whose parents accepted early infant diagnosis	Proportions with 95% CI
Nelson et al., 2012[35]	Lima, Peru	Implementation study in a cohort of TB patients	97%	Proportion of participants that accepted testing	Number of participants; proportion without interval estimation
Newbould et al., 2010[36]	London, UK	Mixed methods study (cohort and survey) with children over one year of age with HIV positive parents	"[Parents all stated that testing their children was highly stressful, and having access to same day results made the process more acceptable." and "The use of POCT was highly acceptable in 100% of families "	Qualitative; proportion of families that considered the use of the POCT acceptable	Qualitative; proportion with no interval estimation
Noble et al., 2012[37]	N/A	Mixed methods study (cohort + survey) with patients of the emergency department	57/57 (100%)	Proportion of participants that accepted testing	Proportions; no interval estimation
Ouladlahsen et al., 2012[38]	University hospital in Casablanca, Morocco	Implementation study in a cohort of patients of the hospital, including children aged > 18 m with unknown serostatus	100%	Acceptance rate	Proportions; no interval estimation
Pai et al., 2008[39]	Department of Obstetrics and Gynecology at the Mahatma Gandhi Institute of Medical Sciences, rural teaching hospital in Sevagram (India)	Mixed methods study (diagnostic evaluation and survey) with women (18–45 y) in active and/or early (incipient) labor	1222/1252 (98%)	Proportion of participants that accepted testing	Proportions; no interval estimation
Parisi et al., 2013[40]	Milan, Italy	Cohort of clients of anonymous testing at different testing facilities	7865/140000 (5.6%)	Proportion of participants that accepted testing and counselling	Proportions; no interval estimation
Ramachandran et al., 2011[41]	Tamilnadu, South India	Cohort of clients attending integrated counseling and testing centers, excluding antenatal women and children	17958 /18329 (98%)	Proportion of participants that accepted testing after counseling	Proportions; no interval estimation
Robbins et al., 2010[42]	Odesa, Kyiv and Donetsk, Ukraine	Cohort of out-of-school youth (15–24 years) in Odesa, Kyiv and Donetsk living part- or full-time on	929/1043 (97%)	Participation rate	Proportions; no interval estimation

		the street			
Ruutel et al., 2012[43]	Tallinn, Estonia	Survey with mostly IDUs and MSM (but not exclusively)	89.1% participation rate; 41/376 declined testing	Participation rate; number of participants that declined testing	Proportions without interval estimation (participation rate); number of individuals
Sattin et al., 2011[44]	Georgia and South Carolina counties	Implementation study in a cohort of ED patients aged 13 to 64 years	8504/ 9343	Proportion of participants that accepted testing	Number of individuals
Scognamiglio et al., 2011[45]	Rome, Italy	Implementation study in a cohort of individuals attending to the mobile unit, usually marginalized people (drug users, sex workers, homeless, immigrants)	323/1028	Acceptance rate, defined as the number of participants that refused testing among those who offered a rapid HIV testing	Proportions; no interval estimation
Stenstrom et al., 2013[46]	St. Paul's Hospital ED, Vancouver, Canada	Survey with adult (19-75 yrs. old) ED patients	1403/2001 (70.1%; 95% CI 68–72)	Proportion of participants that accepted testing	Proportions with 95% CI
Tepper et al., 2009[47]	6 prenatal clinics associated with 6 hospitals from 6 major cities in the US	Diagnostic evaluation among women that were at least 34 weeks gestation and were not in labor and HIV status was unknown	240/266 (90.2%)	Proportion of participants that accepted to participate	Proportions; no interval estimation
Theron et al., 2011[48]	Somerset West district of Western Cape Province, South Africa	RCT in women with unknown serostatus and at least 28 weeks pregnant being admitted for delivery	199/ 542 declined; 343/542 women (63.3%) accepted	Number of participants that declined testing; proportion of participants that accepted enrollment and rapid HIV testing	Number of participants; proportions without interval estimation
van Rooyen et al., 2013[49]	Rural KwaZulu-Natal, South Africa	Cohort of adults	671/739 (91%) consented and were tested; 51/739 (7%) declined	Proportion of participants that consented or that declined participation	Proportions; no interval estimation
Veloso et al., 2010[50]	Public maternity hospitals in Rio de Janeiro and Porto Alegre, Brazil	Diagnostic evaluation; women with unknown HIV serostatus admitted for delivery and infants from HIV-positive mothers	Among pregnant women with unknown HIV serostatus at delivery, 3.3% in Rio de Janeiro (4,347) and 0.3% in Porto Alegre (1,794) refused to participate in this study	Proportion of participants that declined testing	Proportions; no interval estimation
Viani et al., 2013[51]	Tijuana General Hospital in Baja California, Mexico	Diagnostic evaluation with pregnant women	1,383/1,464 (94%) of women in labor/delivery and 1,992/2,075 (96%) of women in prenatal care	Proportion of participants that accepted testing	Proportions; no interval estimation
White et al, 2009[52]	Urban ED in Oakland, California	Cohort of medically stable patients (>= 12 years-old)	18.3%	Proportion of age-eligible patient visits in which HIV screening was offered and accepted	Number of participants; proportion without interval estimation

Young et al., 2013[53]	Mozambique	Implementation study with a cohort of antenatal care patients	83/20646 (0.4%)	Proportion of participants that refused testing	Proportions; no interval estimation

Table1b : Included studies on feasibility measures.

Study	Setting	Study design and population	Outcome	Definitions used by the authors	Reporting
Becker et al., 2013[54]	Health Sciences Centre Hospital (Winnipeg, Manitoba, Canada)	Mixed methods study (diagnostic evaluation and survey) with adults in an ED	96% reported satisfaction with the test and 93% believed it belonged in the ED. 95.5% of participants were confident of the accuracy of the test	Proportion of patients who reported satisfaction with the test or who believed the test belonged in the ED; participants that were confident of the accuracy of the test	Proportions; no interval estimation
Beckwith et al., 2011[5]	Rhode Island Department of Corrections jail, USA	Pilot study with incarcerated inmates; interviews with key informants (nurse, doctors) and focus groups with correctional staff	“Key informant interview and focus group participants overwhelmingly reported positive experiences and opinions about rapid HIV testing at the correctional facility and preferred the rapid testing model to the conventional testing program in place on other days of the week. Benefits were identified at the staff, system, and inmate levels and were frequently related to the use of oral specimens in place of standard phlebotomy."	Improvement in patient cooperation	Qualitative
Burns et al., 2013[8]	Central acute medical admissions unit in London, England	Mixed methods study (implementation and survey) with adults (19–95 yrs. old)	No staff felt that the service had disrupted their job, and all felt that the service should be continued. 92% of doctors believed that more of their own patients were now tested for HIV, and no doctors felt that the service made them less likely to offer a test, with three-quarters believing that the service increased the likelihood of them requesting an HIV test either directly or via the service.	Proportion of the staff that felt that the service had disrupted their job or felt that the service should be continued; proportion of doctors who believe the strategy increased the number of their patients who got tested	Proportions; no interval estimation
Castel et al., 2012[55]	Washington, DC (United States)	Mixed methods (cohort + focus group) with adults testing at outreach events	47% had documented referrals for HIV care and treatment services; quick turnaround times for POCT were mentioned in focus groups, but no actual measurement was performed	Proportion of participants who had documented referrals to HIV treatment and care services among those who screened positive for HIV<br><br>"Participants identified the ease of rapid testing and quick turnaround for obtaining results as incentives for testing. Participants also felt that HIV testing should be a part of any medical examination, similar to getting one’s vital signs checked."	Proportions; no interval estimation
Conners et al., 2012[56]	3 different Veterans Health Administration SUD (substance use disorder) clinics	Mixed methods study (survey and implementation) in a cohort of individuals with substance use disorders	"Facilitators included the ease of NRT integration into workflow, engaged management and an existing culture of disease prevention." and "Findings indicate that NRT can be successfully incorporated into some types of SUD subclinics with minimal	Ease of nurse-initiated rapid test integration into workflow as a facilitator for the implementation of a nurse-initiated testing strategy	Qualitative

	across the USA		perceived impact on workflow and time."		
Gaydos et al., 2013[16]	Johns Hopkins Hospital ED (Baltimore, USA)	Survey with ED patients (18–64 years)	467/473 patients completed the test. 99.8% reported that "overall" the self-test was "easy or somewhat easy" to perform; 96.9% reported they would "probably" or "definitely" test themselves at home if the rapid HIV test were available OTC for purchase	Completion rate; proportion of participants who reported that the self-test was "easy or somewhat easy" to perform	Proportions; no interval estimation
Guenter et al, 2008[57]	Hassle Free Clinic in Toronto, Canada	Cohort of clinic attendees	Scores values: rapid testers would choose the same type of test in the future = 4.78; rapid testers believe test result = 4.66	"Standard testers indicated significantly greater difficulty than rapid testers with the testing procedure. Test counselors also indicated that standard testers had greater difficulty"<br><br>How participants with negative results responded to the question: "I believe that the test result is correct"	Qualitative<br><br>Mean Likert score (on scale from 1 = strongly disagree to 5 = strongly agree)
Manavi et al., 2012[25]	Birmingham Pride event	Cohort of consenting male adults	0.79	Tests per staff-hour carried out	Number of tests
Menacho et al., 2013[30]	Primary care centers in Barcelona, Spain	Mixed methods (survey and cohort); pilot study with adult patients attending to primary care clinics (with an indicator condition vs. without)	Completion of the test: with an indicator condition: 85/85 (100%); controls: 304/313 (97%). Offer rate: 11.5% in the group with indicator condition vs. 5.2% in the control group.	Completion Rate Proportion of those who were offered the test over the total eligible patients	Proportions; no interval estimation
Mungrue et al., 2012[33]	Queen's Park Counselling Centre and Clinic in Trinidad	Mixed methods (diagnostic evaluation + survey) with all persons seeking HIV testing from 2008 at the facility	A friend (127, 51.4%); from the media (72, 29.1%); health care providers (12, 4.8%) were the least common source of information about rapid testing.	How people became aware of rapid testing, defined as the method by which the participants became aware of the existence of rapid testing as a proportion of the total number of participants	Proportions; no interval estimation
Nelson et al., 2012[35]	Lima, Peru	Implementation study in a cohort of TB patients	Health care worker had 1.95 ± 1.62 encounter per patient; 29.7% deferred testing during first encounter of which 93.3% were successfully tested during a follow up visit	Clinical encounters that health care workers had per patient<br><br>Proportion of patients who were successfully tested after having deferred testing in the first encounter	Mean ± SD<br><br>Proportions; no interval estimation
Noble et al., 2012[37]	N/A	Mixed methods study (survey of a cohort of patients of the ED)	"There has been no identifiable negative impact on the ED"	Impact on the ED routine	Qualitative
Nóbrega et al., 2013[58]	Salvador, Brazil	Cohort of pregnant women admitted for delivery at a maternity hospital	13/28 women (46%) received any antiretroviral regimen during pregnancy	Number of infected women that received antiretroviral therapy during prenatal care	Proportions; no interval estimation

Ouladlahsen et al., 2012[38]	University hospital in Casablanca, Morocco	Implementation study in a cohort of patients of the hospital, including children aged > 18 months with unknown serostatus	Annual demand for rapid tests increased from 181 to 540 tests; 19% of the demand came from external clinics; most tests were ordered by doctors from the infectious diseases department (46.8%), followed by reanimation services (18.5%), pneumology (11.8%) and gastroenterology (9.8%)	Which medical specialists ordered HIV tests	Proportions; no interval estimation
Russell et al., 2007[59]	Chuuk State, Micronesia	Survey with residents of remote outer islands (pilot study)	357/370 (96%) of individuals tested returned for post-test counseling	Proportion of individuals tested who returned for post-test counselling	Proportions; no interval estimation
Seewald et al., 2013[60]	Hospital-based methadone program in New York City	Retrospective before-after (before: routine testing; after: targeted testing) study in a cohort of opioid users	Before phase: 1121/7875 (14%) of the patients were tested; after phase: 2700/7870 of the patients (34%) were tested.<br><br>Before phase: 438/1559 tests (28%) were duplicates (i.e. the same individuals were identified and tested two or more times in the same year); after phase: 110/2810 tests (4%) were duplicates	Proportion of patients tested with each strategy	Proportions; no interval estimation
Stenstrom et al., 2013[46]	St. Paul's Hospital ED, Vancouver, Canada	Survey with adult (19-75 yrs. old) ED patients	7.2 minutes (IQR = 4.7–10.1 min)	Time required to perform each test	Median with interquartile ranges
Tepper et al., 2009[47]	6 prenatal clinics associated with 6 hospitals from 6 major cities in the US	Diagnostic evaluation among women that were at least 34 weeks gestation and were not in labor and HIV status was unknown	Time between sample collection and result = 25 minutes (range: 20-110 minutes) vs. 23h (range: 3.5h-45 days) for laboratory-based; 273 (96%) were available within 1 hour. Median test duration: point-of-care = 24 minutes vs laboratory-based testing (35 minutes; P < .0001)	Three different metrics related to TAT: proportion of tests results available within one hour, test duration, and time between sample collection	Proportion of tests available without interval estimation. Test duration: median without range. Time between sample collection: estimate with ranges (unclear whether median or average time was used)
Thomas et al., 2011[61]	Not Given	Mixed methods study (cohort and survey) with MSM	"93% reported they were more likely to undergo repeat screening because of rapid testing. 2% were found to be HIV positive. Of these, 60% cited the rapid test as the primary reason for undergoing screening".	Proportion of participants who said that they were willing to screen repeatedly because of rapid testing; how likely participants would undergo repeat screening because of rapid testing	Proportions; no interval estimation
Veloso et al., 2010[50]	Public maternity hospitals in Rio de Janeiro and Porto Alegre, Brazil	Diagnostic evaluation; women with unknown HIV serostatus admitted for delivery and infants from HIV-positive mothers	48% of women in the study were tested during labor and 51.8% in the postpartum period	Percentage of women tested during labor or in the postpartum period (denominator unclear)	Proportions; no interval estimation

White et al, 2009[52]	Urban ED in Oakland, California	Cohort of medically stable patients above 12 years-old	"HIV screening was offered during 45,159 (38.2%) (...) and completed in 7,923 (6.7%) of the 118,324 ED visits. (...) HIV screening was accepted by patients in 47.9% of the visits in which it was offered, and screening tests were performed in 36.6% of the visits during which patients accepted screening."	Test completion rate per patient visit in the emergency department. Authors also reported another completion rate that was defined similarly as in our framework (of all visits during which patients accepted screening, in how many the test procedure was completed).<br><br>Proportion of visits in which HIV screening was offered and accepted by eligible patients.<br><br>Proportion of patient visits during which HIV testing was offered.	Proportions; no interval estimation

Table 1c. Included studies reporting preference measures.

Study	Setting	Study design and population	Outcome	Definition of acceptability used by the authors	Reporting
Choko et al., 2011[10]	Urban Blantyre, Malawi	Cohort of adult (>= 16 yrs.) members of 60 households and 72 members of community peer groups	260 (91.9%) participants who consented to VCT opted to self-test; the other 23 (8.1%) choose not to self-test. Self-testing at home was the preferred option for future HIV tests for 61.1%.	Proportion of participants who consented to voluntary counselling and testing that also opted to self-test (reported as preference)	Proportion; no interval estimation
Guenter et al, 2008[57]	Hassle Free Clinic in Toronto, Canada	Cohort of clinic attendees	1468/1610 (91%) of participants chose the rapid POC test (versus 8.8% for the standard HIV testing). Among patients with negative test, standard testers were less satisfied overall. 91% of standard testers would choose the same test again vs. 97% of rapid testers.	Proportion of participants who chose the POCT (reported as "quality of experience")	Proportion; no interval estimation
Keller et al., 2011[62]	Two public STI clinics in Baltimore, Maryland	Cohort of clinic attendees	2265/5101 (44%) participants chose the point-of-care test	Proportion of participants who chose the POCT	Proportion; no interval estimation
Marsh et al., 2010[63]	Mobile Clinic in Long Beach, CA, USA	Survey with clients of a mobile testing unit at methadone clinics, substance abuse treatment centers, and a gay and lesbian community center	917/2752 (33%)	Number of rapid tests over the number of total tests performed in a mobile clinic	Proportion; no interval estimation
Mullins et al, 2010[32]	Cincinnati, Ohio	Survey with adolescents recruited at an urban hospital-based adolescent primary care clinic	70% of participants chose a rapid testing method. 50.5% chose the rapid oral fluid test, 30.3% the traditional venipuncture test, and 19.2% the rapid finger stick blood test.	Proportion of participants who chose the POCT	Proportion; no interval estimation
Nelson et al., 2012[35]	Lima, Peru	Implementation study in a cohort of TB patients	Preference for testing site: 54 (58.7%) in their health establishment, 31 (33.7%) at home, 3 (3.3%) in a park, and 4 (4.4%) were tested in another location (i.e., place of work or public place)	Preference for testing site	Proportion; no interval estimation
Ruutel et al., 2012[43]	Tallinn, Estonia	Survey with mostly MSM an IDUs	42.6% preferred fingerpick, 24.2% whole blood draw in a clinical setting, 2.4% saliva rapid testing, 24.9% stated no preference, and 5.9% did not know.	Preference for the HIV test	Proportion; no interval estimation

Table 1d. Included studies reporting measures of disease frequency.

Study	Setting	Study design and population	Outcome	Definition used by the authors	Reporting	Measure classification according to the framework
Anaya et al., 2010[1]	9 shelters of the Los Angeles Homeless Services Authority	Randomized controlled trial with homeless veterans using the shelters	1.50%	HIV prevalence and rate of new incidence	Percentage; no interval estimation	Period prevalence
Benzaken et al., 2011[64]	9 Special Indigenous Health Districts in the Amazon region, Brazil	Cohort of sexually active individuals	3 (0.1%)	Positive HIV tests	Number of individuals who tested positive	Period prevalence
Bucher et al., 2007[7]	San Francisco, USA	Cohort of homeless and marginally housed adults	Seroprevalence was 9.4% (95% CI 7.2–11.5%)	Seroprevalence	Percentage with 95% CI	Period prevalence
Choko et al., 2011[10]	Urban Blantyre, Malawi	Cohort of adult (>= 16 yrs.) members of 60 households and 72 members of community peer groups	18.5% (48 of 260)	HIV prevalence	Proportion; no interval estimation	Period prevalence
Ekouevi et al., 2012[14]	Tokoin Teaching Hospital in Lomé (Togo)	Survey with pregnant women	41/467 (8.8%, 95% CI: 6.4–11.2%)	Prevalence	Proportion with 95% CI	Period prevalence
Ganesan et al., 2010[65]	N/A	Diagnostic evaluation with FSW, MSM, and IDUs	Overall: 3.69% (22/594). FSW: 3.67% (15/408); MSM: 2.95% (4/136); IDU: 6% (3/50).	Samples positive for HIV antibodies	Percentage; no interval estimation	Period prevalence
Gennotte et al., 2013[17]	10 medical centers in Belgium	Cohort of patients with an indicator condition, AIDS-defining illness, or with a characteristic indicating risk for HIV	Seroprevalence: 0 – 0.5% (1/185) in medical centers that delivered care to a 'mixed' population; 5.5% (1/18) in the center that delivered care to patients originating mainly from sub-Saharan Africa. Seroprevalence according to ethnic origin: 0% among Caucasian; 2.2% among Africans; 1.5% among patients with an indicator condition	Seroprevalence	Proportion; no interval estimation	Period prevalence
Guenter et al, 2008[57]	Hassle Free Clinic in Toronto, Canada	Cohort of clinic attendees	HIV prevalence of 2.82% for standard testers and 1.23% for rapid testers (relative risk [RR] 2.3, 95% confidence	HIV prevalence; HIV prevalence by type of test (POCT vs. conventional) and the relative risk	Percentage; no interval estimation	Period prevalence

			interval [CI] 0.82–6.4).	with 95% CI		
Herbert et al., 2012[18]	Hospital for Tropical Diseases, London, UK	Before-after study in a cohort of patients of the open-access emergency clinic	"Almost 1%, with 3 patients from Sub-Saharan Africa and 1 patient from the U.K"	Prevalence of hitherto undiagnosed HIV	Proportion; no interval estimation	Period prevalence
Hoyos et al., 2012[66]	University campuses in Madrid, Málaga and Salamanca (Spain)	Mixed methods study (cohort and survey), supposedly with university students (not specified in the paper)	0.24% (95% CI 0.07–0.62); MSM = 1.6% (95% CI 0,43–3.95)	Global prevalence	Percentage with 95% CI	Period prevalence
Jabbari et al., 2011[20]	Shahid Rajaie, Lengeh, and Shahid Bahonar ports (south of Iran)	Mixed methods (cohort and survey) study with sailors	1/400 (0.25%, 95% CI 0.006%–1.38%)	Study prevalence	Proportion with 95% CI	Period prevalence
Jabbari et al.; 2011[21]	Lavasan (northeast of Tehran, Iran)	Surveillance study with immigrant Afghan population (11 years or older) living in Lavasan, Iran	0.2% (95% CI 0.005–1.2)	Prevalence	Percentage with 95% CI	Period prevalence
Jerene et al, 2007[67]	Arba Minch Hospital in Ethiopia	Adult tuberculosis patients	20.6% (14/68)	HIV prevalence rate	Proportion; no interval estimation	Period prevalence
Kania et.al 2010[22]	Bobo-Dioulasso (Burkina Faso, West Africa)	Diagnostic evaluated among ARV-naïve pregnant women screened for HIV in order to participate in the PMTCT Kesho Bora trial	1.3% (597/44653; 95% CI 1.2 to 1.4)	Prevalence rates of indeterminate and positive serological HIV results	Proportion with 95% CI	Seroprevalence
Levin et al., 2012[23]	Cape Town, South Africa	Diagnostic evaluation among caregivers and previously untested children (aged 17–24 months) attending to immunization clinics	mothers: 21% (107/499)	HIV prevalence (also prevalence of reported HIV exposure) and transmission rate (6-week period)	Proportion (prevalence); rate (transmission)	Period prevalence
Macgowan et al., 2009[24]	Florida, Louisiana, New York, and Wisconsin	Implementation study in a cohort of jail inmates	1.3% (440/33,221); 5.0% among never tested	Reactive rapid HIV tests; prevalence among previously tested and those who had never tested	Proportion; no interval estimation	Period prevalence
Manavi et al., 2012[25]	Birmingham Pride event	Cohort; authors mention only consenting adults (men)	37.7	Incidence	Number of cases per 10000/day	Incidence
Mathe et al., 2008[27]	Rural hospital in North East RDC	Cohort of pregnant women undergoing counselling for HIV (part of a large study examining the risks of postnatal HIV transmission associated with different modes of infant feeding)	1.9% (CI 95 = 1.5–2.5); 9.8% observed with the rapid test	HIV prevalence and seroprevalence used interchangeably	Proportion with 95% CI	Seroprevalence

Melo et al., 2013[28]	Public hospital in Porto Alegre, Brazil	Mixed methods (cohort + survey) study with pregnant women and their partners	"7/1094 previously untested men were identified as HIV+ (prevalence, 0.6%; 95% CI, 0.3%–1.3%)"	Prevalence	Proportion with 95% CI	Period prevalence
Menacho et al., 2013[30]	Primary care centers in Barcelona, Spain	Mixed methods (cohort + survey) study with adult patients attending to primary care clinics with an indicator condition (herpes zoster, seborrheic eczema, mononucleosis syndrome, and leucopenia)	Indicator condition group: 4.7% (95% CI 1.3–11.6) Control (n = 304): 0.3% (95% CI 0.01%–1.82%)	Prevalence	Proportion with 95% CI	Period prevalence
Mikolasova et al., 2013[68]	Hospital in Bunda, Tanzania	Cohort of hospital patients from in- and outpatient units	3–4% prevalence, even among sick patients; after July 2011, monthly prevalence was 10–15 cases/1000–1200 patients (1–1.5%)	Prevalence; incidence (incidence of new cases and monthly prevalence used interchangeably)	Number of cases/number of patients; no interval estimation	Period prevalence; incidence
Morin et al., 2006[69]	12 marketplaces in Epworth and Seke, Zimbabwe	Mixed methods study (cohort and survey) with adults (18 yrs. or older)	321/1099 (29.2%)	Seroprevalence	Proportion; no interval estimation	Period prevalence
Mungrue et al., 2012[33]	Queen's Park Counselling Centre and Clinic in Trinidad	Mixed methods (diagnostic evaluation + survey) with all persons seeking HIV testing from 2008 at the facility	43.7 per 10,000 population in 2009 and 54.6 per 10,000 in 2010; highest seropositivity rate (18.2%) among 20-24 and over 51 yrs. (18.2)	Testing prevalence rate (defined as the number of adults who actually received an HIV rapid test for the two calendar years of complete data); proportion of HIV detected by rapid testing; number of positive tests; seropositivity rate	Proportions; no interval estimation	Period prevalence
Mwembo-Tambwe et al., 2013[70]	Lubumbashi, D.R. Congo	Cohort of pregnant women in labor rooms	21/433 (4.8% IC 95% 3.1%–7.4%); 3.1 % among 15–24 yrs.-old	Prevalence	Proportion with 95% CI	Period prevalence
Ndondoki et al., 2013[34]	Abidjan, Côte d'Ivoire	Survey with children aged 6–26 weeks attending community clinics and their parents/caregivers	Five of the 42 tested infants were infected (11.9%; 95% CI 2.1%–21.7%)	HIV-infected mothers among those who accepted to test	Percentage with 95% CI	Period prevalence
Nóbrega et al., 2013[58]	Salvador, Brazil	Cohort of pregnant women admitted for delivery at a maternity hospital	0.8% (28/3300)	Prevalence and seroprevalence used interchangeably	Proportion; no interval estimation	Seroprevalence

Pai et al., 2008[39]	Department of Obstetrics and Gynecology at the Mahatma Gandhi Institute of Medical Sciences, rural teaching hospital in Sevagram (India)	Mixed methods study (diagnostic evaluation and survey) with women (18–45 yrs.) in active and/or early (incipient) labor	1.23% (95% CI 0.61%–1.8%)	Seroprevalence	Proportion with 95% CI	Seroprevalence
Parisi et al, 2013[40]	Milan, Italy	Cohort of adults unaware of their serostatus	50/7865 (0.63%)	Oral test reactive	Proportion; no interval estimation	Period prevalence
Portman et al., 2013[71]	Northern England (UK HIV Testing week)	General public cohort	2/94 (2.1%)	Positivity rate / reactive tests	Proportion; no interval estimation	Period prevalence
Qvist et al., 2014[72]	Copenhagen, Denmark	Cohort of MSM	1% of all rapid tests performed; 11% of all new cases among MSM in Copenhagen; 15/539 (3%) positive tests in 2011-2012	Proportion of positive tests over all tests done; proportion of new cases detected with POCT over all new cases in that specific population; HIV-positive rate	Proportion; no interval estimation	Period prevalence
Ramachandran et al., 2011[41]	Tamilnadu, South India	Cohort of clients attending integrated counseling and testing centers, excluding antenatal women and children	"overall HIV seroprevalence was 4.1% (varied from 2.6 to 6.2% in different districts)"	Seroprevalence	Percentage; no interval estimation	Seroprevalence
Robbins et al., 2010[42]	Odesa, Kyiv and Donetsk, Ukraine	Cohort of out-of-school youth (15–24 years) living part- or full-time on the street	"Overall HIV seroprevalence was 18.4% (95% CI: 16.2 – 20.2) "	Seroprevalence	Percentage with 95% CI	Seroprevelance / period prevalence
Ruutel et al., 2012[43]	Tallinn, Estonia	Survey with mostly IDUs and MSM (but not exclusively)	58/308	Preliminary positive cases identified	Proportion; no interval estimation	Period prevalence
Theron et al., 2011[48]	Somerset West district of Western Cape Province, South Africa	Cluster randomized trial in women with unknown serostatus and at least 28 weeks pregnant being admitted for delivery	"13.1% (45/343) 95% CI (9.7–17.2)"	Seroprevalence and prevalence used interchangeably	Percentage with 95% CI	Seroprevalence
van Rooyen et al., 2013[49]	Rural KwaZulu-Natal, South Africa	Cohort of adults	30%	Proportion of individuals who tested positive	Percentage; no interval estimation	Period prevalence
Veloso et al., 2010[50]	Public maternity hospitals in Rio de Janeiro and Porto Alegre, Brazil	Diagnostic evaluation; women with unknown HIV serostatus admitted for delivery and infants from HIV-positive mothers	Porto Alegre = 6.5% (N=1,439); Rio de Janeiro = 1.3% (N=3.778)	Prevalence	Proportion; no interval estimation	Seroprevalence

Viani et al., 2013[51]	Tijuana General Hospital in Baja California, Mexico	Diagnostic evaluation with pregnant women	Tested during labor and delivery: (19/1,383, 1.37%, 95% CI 0.85%–2.18%; during prenatal care: (5/1992, 0.25%, 95% CI 0.09%–0.62%)	Seroprevalence among women tested during labor/delivery or during prenatal care; prevalence	Proportion with 95% CI	Seroprevalence
Young et al., 2013[53]	Mozambique	Implementation study with a cohort of antenatal care patients	"Routine PMTCT results was 13.5% (IQR 7.0-21.4%]; site-level prevalence estimates calculated from surveillance data was 14.4% (IQR 8.2-21.8%)."	Prevalence	Percentage with IQR (site-level prevalence)	N/A

Table 1e. Included studies reporting impact measures.

Study	Setting	Study design and population	Outcome Reported	Definitions used by the authors	Reporting
Anaya et al., 2010[1]	9 shelters of the Los Angeles Homeless Services Authority	Randomized controlled trial with homeless veterans using the shelters	"Testing rates were 100.0% in rapid testing (RT) arm and 3.3% in referral arm (P < .0001). Test result receipt rates were 98.5% in RT arm and 0.0% in Referral arm (P < .0001)"	Testing rate (proportion of participants tested in each arm); increase in testing not calculated<br><br>Number of results received (documented in both arms: rapid testing and referral to conventional testing	Proportion; no interval estimation
Anaya, 2008[73]	2 Veteran Affair Health Care Sites in Southern California; one was a hospital and other was outpatient clinic serving indigent and homeless veterans	Randomized controlled trial evaluating three models of HIV testing among adults (between 18–65 yrs.) unaware of HIV status	Individuals in model A (RR= 2.06 95% CI= 1.1-3.7) were less likely to receive results than in C (RR=5.2; 95%CI = 3.1–8.9) or B (RR=2.25; 95% CI = 1.82–3.58)	Likelihood of receiving test result<br><br>A=traditional HIV counseling/testing;<br><br>B= Nurse-initiated screening +traditional counseling/testing;<br><br>C= Nurse-initiated + streamlined counseling/rapid testing	Relative risk with confidence intervals
Ashby et al., 2010[3]	United Kingdom	Survey with patients of a polyclinic serving a highly migrant urban population	27/71 (38%) of those tested had at least 1 identifiable risk factors and of those, 17/27 (63%) had never tested; no new diagnoses of HIV were made.	Number of tests performed on participants with at least one identifiable risk factor for HIV<br><br>Number of participants never tested before; number of new diagnosis	Proportion; no interval estimation<br><br>Number (no new cases were found)
Batey et al., 2012[4]	Alabama, USA	Interviews with individuals presenting to a Level I trauma and academic medical center in Alabama	No new positives detected	Number of new positives	Number (no positives were found)
Beckwith et al., 2010[74]	Rhode Island Department of Corrections central jail facility	Cohort of incarcerated males	28% (30/108) of those who completed the follow-up visit received results from their standard HIV test conducted with no significant difference between the rapid (31%) and standard testing (26%) groups (P = 0.29)	Number of results received	Proportion; no interval estimation
Beckwith et al., 2011[5]	Rhode Island, USA	Rhode Island Department of Corrections inmates; interviews with key informants (nurse, doctors) and focus groups with	12 (0.8%) rapid HIV tests with oral specimens were reactive; 1 new diagnose. 8 disclosed HIV-positive status after rapid testing; 3 with chronic HIV infection did not disclose.	Number of reactive tests; number of new diagnosis; number of positive results disclosed by the participants	Proportion without interval estimation; number;

		correctional staff	No new positives detected.		number
Benzaken et al., 2011[64]	9 Special Indigenous Health Districts in the Amazon region, Brazil	Cohort of sexually active individuals	38799/83311 (47%)	Test uptake (defined as the proportion of participants tested for HIV	Proportion; no interval estimation
Bowles et al., 2008[6]	Boston; Chicago; Detroit; Kansas City, Missouri; Los Angeles; San Francisco; and Washington, D.C.	Cohort of adults with unknown serostatus	331/23900 (1.4%) received preliminary positive HIV test results; 267/23900 (1.1%; range 0.5% to 1.8%) newly diagnosed HIV infections. 75% received their confirmatory test results. 30% of participants had never been tested for HIV, and of those who had, 43% had not been tested in the past year. 64% were referred to care	Number of preliminary results received; proportion of positives who received confirmatory results; number of newly diagnosed infections; number of first time testers; number of participants not tested in the past year; proportion of participants referred to care	Proportion; no interval estimation; proportion with range; proportion with no interval estimation
Bucher et al., 2007[7]	San Francisco, USA	Cohort of homeless and marginally housed adults	30 newly diagnosed cases with confirmatory results, of whom 26 (86.7%; 95% CI 69.3–96.2) reported at least one contact with a primary healthcare provider in the 6 months following diagnosis; 174/1213 (14.3%) had never been tested before. 7/37 newly diagnosed were lost to follow up	Number of positive tests with confirmatory results; number of participants reporting at least one contact with a HCP in 6 months following diagnosis<br><br>Loss to follow-up	Number<br><br>Proportion with 95% CI; proportion with no interval estimation
Burns et al., 2013[8]	Central acute medical admissions unit in London, England	Mixed methods study (implementation and survey) with adults (19-95 yrs. old)	3/135 tests (2.2%) were reactive on POCT and all were confirmed by laboratory testing. All three patients were seen by specialist HIV services while in-patients and remained engaged with HIV services 12 months on. Only one of the three had previously been tested for HIV, over 5 years previously.	Reactive tests; confirmed reactivity;<br><br>Number of participants with positive tests who have been previously tested during the last 5 years; number of patients who were seen by specialist in HIV services; number of patients who remained engaged in care after 12 months	Proportion; no interval estimation<br><br>Number
Carballo-Diéguez et al., 2012[9]	New York City, USA	Mixed methods study (diagnostic evaluation and survey) with MSM	"Lack of partner resistance to taking the test was seen as a good sign. When partners resisted, participants often interpreted it as a warning not to have sex with that person."	Partner resistance to test when offered the home-based test as reported by the participants	Qualitative
Choko et al., 2011[10]	Urban Blantyre, Malawi	Cohort of adult (>=16 y) members of 60 households and 72 members of	After self-testing, all participants would recommend self-testing to friends and	Proportion of participants that would recommend self-testing to others	Proportion; no interval

		community peer groups	family		estimation
Cirone et al., 2013[11]	Urban level 1 trauma center (presumably at the US)	Cohort of ED patients (18-64 yrs.); pilot study	None of the tests were reactive<br><br>50% of the patients had not previously received an HIV test.<br><br>Average test time: 24.5 minutes	Number of negative tests<br><br>Number of patients previously untested<br><br>Test time	Number<br><br>Proportion; no interval estimation<br><br>Average; no measure of dispersion
Criniti et al., 2009[12]	Ambulatory obstetrics and gynecology clinic, Philadelphia, PA	Survey with women (18-45 yrs.) at 32 weeks or more of gestation and a documented HIV negative test before 24 weeks of gestation	No new cases	Number of new cases	Number (no new cases were found)
Darling et al., 2012[13]	Red Lights District in Lausanne, Switzerland	Survey with clients of FSW (all were men)	No reactive test; 52–71% had never undergone an HIV test	Number of reactive tests; number of participants who ever tested before	Number (none of the tests was reactive); Proportion with no interval estimation
Ekouevi et al., 2012[14]	Tokoin Teaching Hospital in Lomé (Togo)	Survey with pregnant women	14/41 (34.1%) women living with HIV were newly diagnosed in labor room; 41.5% (n=17) of the 41 women living with HIV had not initiated any PMTCT intervention antenatal.<br><br>"Among the 41 women diagnosed as living with HIV during labour, 34% (14 women) had not been tested for HIV during pregnancy and were missed opportunities.”	Newly diagnosed individuals; number of PMTCT initiated; missed opportunities (women who had not been tested during pregnancy)	Proportion; no interval estimation
Garrard et al., 2010[15]	United Kingdom	Implementation study in a cohort of women attending a termination of pregnancy service were recommended HIV tests as part of routine consultation	"Of those where results were available, 0.52% (5/972) were newly diagnosed HIV positives"	Number of newly diagnosed individuals out of those with results available	Proportion; no interval estimation

Guenter et al, 2008[57]	Hassle Free Clinic in Toronto, Canada	Cohort of clinic attendees	"Among the rapid testers, 100% received an initial result, and 18 of 22 testing positive returned for confirmatory results."	Number of results received<br><br>Number of preliminary positive results	Proportion; no interval estimation
Herbert et al., 2012[18]	Hospital for Tropical Diseases, London, UK	Implementation study in a cohort of patients of the open-access emergency clinic	"Testing rates increased; with 6.8% tested in the pre-universal testing period, to 44.8% in the universal POCT period (p <0.0001)";<br><br>"No new HIV positive patients were identified in the pre-screening period, 2 with laboratory screening and 2 with POCT (laboratory confirmed)."	Testing rate (proportion of participants tested with or without POCT); increase in testing not calculated<br><br>Number of newly diagnosed patients identified in the pre-screening period, with laboratory screening or with POCT (laboratory confirmed)	Proportion; no interval estimation<br><br>Number
Hernandez et al., 2013[75]	Urban Level-1 Trauma Center	Adult patients who had STD testing at the Emergency Department	2.3 hours in the patients receiving an HIV test, compared to 2.6 hours in the non-tested patients (p = 0.42)	Length of stay in the Emergency Department	Median time with p-statistic
Hooshyar et al., 2014[19]	Dallas, Fort Worth, and Texoma (Texas, USA)	Cohort of homeless veterans attending to an outreach event	92% of the tested veterans obtained their test results at the events (all negative)	Number of participants that received their results at the testing event	Proportion; no interval estimation
Hoyos et al., 2012[66]	University campuses in Madrid, Malaga and Salamanca (Spain)	Not specified, but appears to be university students (cohort)	78,4% tested for the first time; 5/1668 reactive tests (4 confirmed new cases);	Number of first time testers; number of reactive tests	Proportion; no interval estimation
Hoyos et al., 2013[76]	Spain (Madrid City, two working-class suburbs of Madrid, three coastal cities in the southeast, and in the Canary Islands)	Mixed methods study (cohort and survey) with MSM, men who have sex with women, and women born in Spain or Latin-America	2455/5920 (40%) had never undergone an HIV test before	Number of first time testers	Proportion; no interval estimation
Jabbari et al., 2011[21]	Lavasan (northeast of Tehran, Iran)	Surveillance study with immigrant Afghan population (11 years or older) living in Lavasan, Iran	1 positive result confirmed as positive; 5 indeterminate results	Number of positive results confirmed by a second tests; number of indeterminate test results	Numbers
Keller et al., 2011[62]	Two public STI clinics in Baltimore, Maryland	Cohort of clinic attendees	"34 truly newly diagnosed patients, 16 used POC testing"<br><br>27 (47%) engaged in care	Number of "truly" newly diagnosed individuals with HIV<br><br>Number of participants engaging in care, defined as 2 clinic visits for HIV care after post-test counselling within 6 months of initial positive test result	Number<br><br>Proportion; no interval estimation

Levin et al., 2012[23]	Cape Town, South Africa	Diagnostic evaluation among caregivers and previously untested children (aged 17–24 months) attending to immunization clinics	No previously unknown HIV infection was detected	Number of previously unknown infections	Number (no previously unknown infections were found)
Macgowan et al., 2009[24]	Florida, louisiana, New York, and Wisconsin	Implementation study in a cohort of jail inmates	>99.9% received test results. 269 (0.8%) confirmed new diagnoses (1.3% HIV+ with rapid tests; 5.0% with rapid tests among never tested); newly diagnosed represented 64% of those with reactive tests.	Number of results received<br><br>Proportion of confirmed new diagnosis overall results; number of new diagnosis with rapid tests	Number<br><br>Proportion; no interval estimation
Manavi et al., 2012[25]	Birmingham Pride event	Cohort; authors mention only consenting adults (men)	6/405 (1.5%) were diagnosed with HIV infection; only 1 was already aware of his HIV status	Number of HIV diagnosis; number of individuals that were already aware of their positive serostatus	Proportion without interval estimation; number
Martin et al., 2011[26]	24 sites in New Jersey	Diagnostic evaluation with clients of health facilities in the rapid testing program	"99.95% of all screened clients were resolved by the end of the initial screening visit. Only the 32 discordants (0.062%) required additional testing and delays before reaching a final result and being potentially linked to care."<br><br>289/394 (73.3%) positives by rapid testing had appointments made with a healthcare provider during the first visit	Number of patients screened and "resolved" by the end of the initial screening visit; number of test discordants requiring further testing<br><br>Number of rapid test positives who had appointments with a HCP during the first visit	Proportion; no interval estimation
Mayhood et al., 2008[77]	Northern Tanzania	Mixed methods study (diagnostic and cost evaluation) with clients of the KIWA KKUKI clinic (Women Against AIDS in Kilimanjaro)	"1,938/12,737 (15.2%) were concordant positive; 10,736/12,73 (84.3%) were concordant negative; 63/12,73 (0.5%) discordant rapid HIV test results	Number of positive or negative concordant results; number of discordant results	Proportions; no interval estimation
Mehta et al., 2008[78]	Boston, USA	Cohorts of patients	"Among 16,750 HIV tests, 258 (1.5%) were positive. Thus, 229 (1.37%; 95% confidence interval [CI] 1.20, 1.56) of 16,696 patients were newly diagnosed with HIV"<br><br>"Of the 258 patients who tested HIV positive, 29 reported a previous HIV-positive test result in their pretest	Number of positive results; number of newly diagnosed individuals<br><br>Number of patients with a previous HIV-positive test result in their pretest counseling assessment	Proportions (95% confidence intervals provided for newly diagnosed participants)

			counseling assessment"		Number
Melo et al., 2013[28]	Public hospital in Porto Alegre, Brazil	Mixed methods (cohort + survey) study with pregnant women and their partners	4/2888 new infections among women; uncovered 14/1101 serodiscordant couples	Number of new infections; number of serodiscordant couples uncovered	Number
Melvin et al., 2004[29]	Instituto Materno-Perinatal in Lima, Peru	Diagnostic evaluation with previously untested pregnant women presenting to the emergency room that were not beyond the first stage of labor	80.7% previously untested. Number treated unclear (21/23 treated reported in the results; 11/12 of women tested during labor and 7/10 of women tested earlier reported as treated in the discussion<br><br>25 women tested HIV seropositive by both rapid tests, but 2/25 (8%) failed to confirm HIV-1 or -2 seropositivity by EIA	Number of previously untested participants; Number treated (different proportions of participants treated given throughout the paper)<br><br>Number of participants with positive results in both rapid tests	Proportion; no interval estimation<br><br>Number
Metsch et al., 2012[79]	Community treatment programs for drug or alcohol abuse in several sites across the United States	Randomized controlled trial with HIV-negative (or unknown status) adults who reported no past-year HIV testing	"Participants randomized to on-site rapid testing were significantly more likely to complete and receive the results of an HIV test compared with participants randomized to the off-site referral arm (P < .001; aRR = 4.52; 97.5% confidence interval [CI] = 3.57, 5.72)"; "Offering HIV rapid testing on site in drug treatment centers increased receipt of test results and uptake."	Likelihood of receiving test result; number of reactive results received; likelihood of complete testing procedure (POCT compared to conventional testing)	Adjusted relative risk with confidence intervals; number of reactive results
Mikolasova et al., 2013[68]	Hospital in Bunda, Tanzania	Cohort of hospital patients from in- and outpatient units	194 new cases of HIV were detected (in 2005–2010) and 226 (19%) of these patients receive antiretroviral therapy (ARV): 20 new treated cases/month and 240 new treated cases/year	Number of new cases detected<br><br>Number of new cases that received ART	Number<br><br>Proportion without interval estimation; number of cases per month or year

Mkwanazi et al. 2008[31]	8 rural clinics in KwaZulu-Natal (South Africa)	Cohort of pregnant women undergoing counselling for HIV (part of a large study examining the risks of postnatal HIV transmission associated with different modes of infant feeding)	Women were more likely to return for results before rapid tests were introduced than after: 65% (2800/4321) vs. 50% (2321/4368), with declining trend over time for returning for results: those testing in 2002 (OR 0.61; 95% CI 0.46–0.81), 2003 (OR 0.23; 95% CI 0.18–0.31; p < 0.001) and 2004 (OR 0.32; 95% CI 0.24–0.42) were less likely to return for results compared to those testing in 2001	Likelihood of receiving test result by introduction of rapid test and by year	Proportion without confidence intervals<br><br>Odds ratio with confidence intervals
Mullins et al., 2010[32]	Cincinnati, Ohio	Survey with adolescents were recruited from an urban hospital-based adolescent primary care clinic	22/40 (55%)	Number of testers who returned for results (some participants preferred to come back on another day to pick up their results, even though they were tested with a POCT)	Proportion; no interval estimation
Mungrue et al., 2012[33]	Queen's Park Counselling Centre and Clinic in Trinidad	Mixed methods (diagnostic evaluation + survey) with all persons seeking HIV testing from 2008 at the facility	"The prevalence of HIV testing among [adults] (...) who actually received an HIV rapid test for the two calendar years of complete data (ie, 2009 and 2010) was 43.7 per 10,000 population in 2009 and 54.6 per 10,000 in 2010". Also report "proportion of HIV detected by rapid testing" by gender without confidence intervals. Highest seropositivity rate (18.2%) among 20-24 and over 51 yrs (18.2%). No overall prevalence reported."<br><br>"Over the study period, the number of persons who received rapid tests increased"<br><br>45 positive tests, 44 confirmed	Prevalence of HIV testing; increase in the number of tests performed; increase in the number of participants who received their results<br><br>Increase in the number of participants who received their results<br><br>Number of positive tests; number of positive tests confirmed	Number of tests; number of tests per 10000 population per year<br><br>Qualitative;<br><br>Number
Ndondoki et al., 2013[34]	Abidjan, Côte d'Ivoire	Survey with children aged 6–26 weeks attending community clinics and their parents/caregivers	81 mothers were identified as HIV infected (4.5%; 95% CI 3.5%–5.4%); 54 new cases detected. 2 fathers were HIV+ (5.7%; 95% CI 0–13.4%).	Number of mothers identified as infected; number of new cases detected; number of fathers infected	Proportions with 95% CI; number of new cases
Nelson et al., 2012[35]	Lima, Peru	Implementation study in a cohort of TB patients	"All results were communicated to the health provider of total participants, including 100% among HIV-positive patients."	Number of test results received when rapid test was offered (results received by testing site and compared to conventional testing)	Proportion; no interval estimation

Noble et al., 2012[37]	N/A	Mixed methods study (cohort + survey) with patients of the emergency department	5/57 (8.8%) tests were reactive; all confirmed	Number of reactive tests with confirmed results	Proportion; no interval estimation
Ouladlahsen et al., 2012[38]	University hospital in Casablanca, Morocco	Implementation study in a cohort of patients of the hospital, including children aged > 18 months with unknown serostatus	180/1105 (16.3%) positives, of which 98.9% confirmed by WB. Rapid tests allowed change of treatment for 12 cases of dyspneic pneumopathy and 7 of brain abscess that were being treated as pneumocyst and toxoplasmose, respectively; 100% HIV+ were linked to care with average CD4 = 280-320 cells/uL	Number of positive tests; proportion of confirmed tests over initial positives<br><br>Treatment change in response to test result<br><br>Number of participants linked to care	Proportion; no interval estimation<br><br>Number<br><br>Proportion; no interval estimation
Pai et al., 2008[39]	Department of Obstetrics and Gynecology at the Mahatma Gandhi Institute of Medical Sciences (MGIMS), rural teaching hospital in Sevagram (India)	Mixed methods study (diagnostic evaluation and survey) with women (18–45 y) in active and/or early (incipient) labor	659/1222 participants (54%) were never tested before during their pregnancy. 1003/1222 women 1003 (82%; 95% CI 79.8%–84.2%) had never been HIV tested, or had been tested but were unaware of their HIV status.<br><br>11/15 HIV infected (73.3%; 95% CI 47.5%–90.9%) women were newly diagnosed at point-of-care and 14/15 (93.3%; 95% CI 71.3%–99.7%) received PMTCT interventions.<br><br>Time between eligibility assessment and informed consent = 5–10 min; time for pretest counseling = 15 min, and time for rapid testing = 20 min.<br><br>Total time to referral for PMTCT intervention = 40–60 min.<br><br>75% (12/15) HIV+ women discussed their serostatus with their husbands and the remainder (3/15) with their mothers.	Number of women never tested before during pregnancy; never tested or tested but unaware of serostatus<br><br>Number of participants with new infections<br><br>Number of participants who received interventions<br><br>Time for pretest counselling; time taken to perform the test; total time to referral to PMCT intervention<br><br>Proportion of infected women who discussed their serostatus with their partners or with their mothers	Proportion (only never treated or tested but unaware with 95% CI)<br><br>Proportion with 95% CI<br><br>Proportion with 95% CI<br><br>Unclear if median or average; total time to referral was reported with range<br><br>Proportion; no interval estimation
Parisi et al., 2013[40]	Milan, Italy	Cohort of clients of anonymous testing at different testing facilities	0.6% in total with a positive test; 50 new infections; 48% had never undergone an HIV screening test	Number of subjects with positive saliva test<br><br>Number of new infections<br><br>Number of participants who had previously tested	proportion without interval estimation; Number; proportion

					without interval estimation;
Puro et al., 2004[80]	St Anna University of Ferrara (hospital A) and the University Hospital of Perugia (hospital B), public hospitals in Italy.	Cohort of patients that were a source of HCW occupational exposure to HIV	Reduction of HCWs who were given unnecessary PEP (84 to none in one hospital; 93 to none in another)	Decrease in unnecessary PEP (post exposure prophylaxis)	Number and difference after POCT use
Qvist et al., 2014[72]	Copenhagen, Denmark	Cohort of MSM	Before phase: 3/1121 patients (0.27%; 95% CI 0.13–0.52) were newly diagnosed with HIV; all 3 received rapid test results, but only 1/3 received confirmatory result and 0/3 adhered to first HIV medical appointment. After phase: 2700/7870 patients (34%) were tested; 8/2700 (0.29%) were newly diagnosed with HIV. All 8 received confirmatory blood test result; 5/8 adhered to first HIV medical appointment. 63% of 1510 tests performed (in 2011-2012) were retests	Number of newly diagnosed individuals<br><br>Newly diagnosed individuals who received confirmatory results<br><br>Number tested for the first time at the mobile clinic; number of retests<br><br>Number of patients who adhered to first medical appointment	Proportions with 95% CI<br><br>Proportion; no interval estimation<br><br>Proportion; no interval estimation<br><br>Proportion; no interval estimation
Robbins et al., 2010[42]	Odesa, Kyiv and Donetsk, Ukraine	Cohort of out-of-school youth (15–24 years) in Odesa, Kyiv and Donetsk living part- or full-time on the street	85%	Participants with positive results who did not reported a previous HIV diagnosis	Proportion; no interval estimation
Ruutel et al., 2012[43]	Tallinn, Estonia	Survey with mostly IDUs and MSM (but not exclusively)	58 received a positive test result during this study (46 were new cases, 30 reported no previous HIV test)	Number of positive results; number of new cases; number of participants who reported no previous test	Number
Sattin et al., 2011[44]	Georgia and South Carolina counties	Implementation study in a cohort of ED patients aged 13 to 64 years	41 reactive tests (35 confirmed positive). Nearly 75% of patients confirmed as HIV positive kept their first HIV clinic appointment.	Number of reactive tests; number of confirmed results<br><br>Number of participants with positive tests who kept their first clinic appointment	Number<br><br>Proportion; no interval estimation
Schulden et al., 2008[81]	Miami Beach, Florida, New York City, and San Francisco	Survey with self-identified transgender who were at least 13 years of age and who were not known to be infected with HIV were recruited	67 (12%) newly diagnosed	Number of newly diagnosed individuals	Proportion; no interval estimation

Scognamiglio et al., 2011[45]	Rome, Italy	Implementation study in a cohort of individuals attending to the mobile unit, usually marginalized people (drug users, sex workers, homeless, immigrants)	For 43% of cases this was the first approach to HIV testing	Number of participants to whom it was the first approach to test<br><br>Authors reported a "high proportion of failure to return for confirmatory testing", but no number was provided	Proportion (only never treated or tested but unaware with 95% CI)<br><br>Qualitative
Seewald et al., 2013[60]	Hospital-based methadone program in New York City	Retrospective before-after (before: routine testing; after: targeted testing) study in a cohort of opioid users	Three of the 1121 (0.27%; 95% CI 0.13–0.52) were newly diagnosed with HIV. 3/3 HIV-positive received their rapid test results, only 1/3 received confirmatory blood test, and none adhered to their first HIV medical appointment	Number of newly diagnosed individuals<br><br>Number of positive rapid test results received; proportion of rapid test positives who received confirmatory results<br><br>Number of patients who adhered to first medical appointment	Proportions with 95% CI<br><br>Proportion; no interval estimation
Tepper et al., 2009[47]	6 prenatal clinics associated with 6 hospitals from 6 major cities in the US	Diagnostic evaluation among women that were at least 34 weeks gestation and were not in labor and HIV status was unknown	7 women delivered before laboratory results were available; no women delivered before rapid test result. Time between sample collection and result = 25 min (range: 20-110 min) vs. 23h (range: 3.5 h–45 days) for laboratory-based; 273 (96%) were available within 1 hour.<br><br>Median test duration: point-of-care = 24 min vs laboratory-based testing (35 min).	Number of cases in which results didn't arrived in time for PMTCT<br><br>Time between sample collection and result; results available within 1 hour; test duration	Number<br><br>Results within 1 hour as proportions without interval estimation; other measures reported as median with or without range
Theron et al., 2011[48]	Somerset West district of Western Cape Province, South Africa	RCT in women with unknown serostatus and at least 28 weeks pregnant being admitted for delivery	34 babies returned for follow up. In intrapartum arm: all women in true labor received their test results before delivery; 18/19 newborns exposed to HIV in the intrapartum arm were prophylactically treated with NVP and AZT prior to hospital discharge, and the treatment was initiated within 12 h of birth in 17 of these newborns (89.5%). Postpartum arm: treatment was initiated within 12 h of birth in 21/26 newborns (81%) prophylactically treated with NVP and AZT	Number of babies positive for HIV among those who returned for follow-up<br><br>Number of results arrived in time; number of newborns treated prior discharge and within 12 h of birth<br><br>Number of results received in time	Number<br><br>Proportions; no interval estimation<br><br>Proportions; no interval estimation

Thomas et al., 2011[61]	N/A	Mixed methods study (cohort and survey) with MSM	2% HIV positive.<br><br>10% had never been tested previously	Number of participants found to be positive<br><br>Number of previously untested participants	Proportion; no interval estimation
van Rooyen et al., 2013[49]	Rural KwaZulu-Natal, South Africa	Cohort of adults	214/739 (32%) were tested for the first time.<br><br>201/739 (34%; IQR 27–43) HIV-infected participants were identified PCT; 73/201 (36%) newly diagnosed.<br><br>48/58 couples of which both partners were tested for HIV had concordant serostatus (43 concordant negative and 5 concordant positive); 10/58 were serodiscordant.<br><br>Linkage to care: 197/201 (99%), 199/201 (100%), and 196/201 (100%) completed their months 1, 3, and 6 follow-up visits, respectively. Cumulative probability of linkage to care: 57% at baseline, 96% after 6 months.<br><br>Cumulative probability of ART initiation by 3 months: 86%. 36 participants initiated ART during the study.<br><br>3/201 HIV+ died during the study	Number of participants tested for the first time<br><br>Number of HIV-infected participants identified; number of newly diagnosed; number concordant (positive or negative) couples and of serodiscordant couples<br><br>Number of patients who completed 1, 3, and 6 months' follow-up visits; linkage to care<br><br>Treatment initiation<br><br>Number of deaths among study participants	Proportion; no interval estimation<br><br>Proportions; interquartile range reported for the proportion of HIV-infected participants identified<br><br>Proportion without interval estimation; cumulative probability<br><br>Cumulative probability; number of participants who initiated ART<br><br>Number

Veloso et al., 2010[50]	Public maternity hospitals in Rio de Janeiro and Porto Alegre, Brazil	Diagnostic evaluation; women with unknown HIV serostatus admitted for delivery and infants from HIV-positive mothers	143/5217 (2.7%) positive results<br><br>100% of newborns whose mothers were tested in the postpartum received post-exposure ZDV; 33.3% of them were not breastfed in Rio de Janeiro and none was breastfed in Porto Alegre.<br><br>Exposure to breast milk was completely avoided for 96.8% and 51.1% of cases in Porto Alegre and Rio de Janeiro, respectively.	Number of positive results<br><br>PMTCT initiated; prevention of exposure to breast milk	Proportion; no interval estimation
Viani et al., 2013[51]	Tijuana General Hospital in Baja California, Mexico	Diagnostic evaluation with pregnant women	214 (32%) were tested for the first time with 73 (36%) new diagnoses.<br><br>26 women had positive parallel rapid HIV testing (24 tested positive by confirmatory WB).<br><br>"Of the HIV-infected participants, 197 (99%), 199 (100%), and 196 (100%) completed their months 1, 3, and 6 follow-up visits, respectively."	Number of participants tested for the first time; Number of new diagnosis; number of diagnosis confirmed<br><br>Number of patients who completed 1, 3, and 6 months' follow-up visits	Proportion without interval estimation; number
White et al, 2009[52]	Urban ED in Oakland, California	Cohort of medically stable patients above 12 years-old	56 (0.7%) positive test; 90 (89.1%) patients with newly diagnosed HIV infection linked to follow up	Number of positive screening rapid test; number of patients linked to follow-up	Proportion; no interval estimation

References

1. Anaya H, Feld J, Hoang T, Knapp H, Asch S. Implementing an HIV rapid testing intervention for homeless veterans in shelter settings within Los Angeles county. *Journal of the International Association of Physicians in AIDS Care* 2010; **9 (1)**: 47.
2. Arfai N, Squires K, Ezeala Y, et al. Demographics and risk factors of patients who decline opt-in hiv screenings in an urban emergency department. *Ann Emerg Med* 2011; **1)**: S200.
3. Ashby J, Braithewaite B, Walsh J, Gnani S, Fidler S, Cooke G. HIV testing uptake and acceptability in an inner city polyclinic. *AIDS Care* 2012; **24**(7): 905-9.
4. Batey DS, Hogan VL, Cantor R, et al. Short communication routine HIV testing in the emergency department: assessment of patient perceptions. *AIDS Res Hum Retroviruses* 2012; **28**(4): 352-6.
5. Beckwith CG, Bazerman L, Cornwall AH, et al. An evaluation of a routine opt-out rapid HIV testing program in a Rhode Island jail. *AIDS Educ Prev* 2011; **23**(3 Suppl): 96-109.
6. Bowles KE, Clark HA, Tai E, et al. Implementing rapid HIV testing in outreach and community settings: results from an advancing HIV prevention demonstration project conducted in seven U.S. cities. *Public Health Rep* 2008; **123 Suppl 3**: 78-85.
7. Bucher JB, Thomas KM, Guzman D, Riley E, Dela Cruz N, Bangsberg DR. Community-based rapid HIV testing in homeless and marginally housed adults in San Francisco. *HIV Med* 2007; **8**(1): 28-31.
8. Burns F, Edwards S, Woods J, et al. Acceptability, feasibility and costs of universal offer of rapid point of care testing for HIV in an acute admissions unit: Results of the RAPID project. *HIV Medicine* 2013; **14**(SUPPL.3): 10-4.
9. Carballo-Dieguez A, Frasca T, Balan I, Ibitoye M, Dolezal C. Use of a rapid HIV home test prevents HIV exposure in a high risk sample of men who have sex with men. *AIDS Behav* 2012; **16**(7): 1753-60.
10. Choko AT, Desmond N, Webb EL, et al. The uptake and accuracy of oral kits for HIV self-testing in high HIV prevalence setting: a cross-sectional feasibility study in Blantyre, Malawi. *PLoS Med* 2011; **8**(10): e1001102.
11. Cirone MV, Probst BD, Stake CE, et al. The implementation of opt-in rapid HIV testing in an urban emergency department. *Annals of Emergency Medicine* 2013; **1)**: S64.
12. Criniti SM, Aaron E, Levine AB. Using the rapid HIV test to rescreen women in the third trimester of pregnancy. *J Midwifery Womens Health* 2009; **54**(6): 492-6.
13. Darling KE, Diserens EA, N'Garambe C, et al. A cross-sectional survey of attitudes to HIV risk and rapid HIV testing among clients of sex workers in Switzerland. *Sex Transm Infect* 2012; **88**(6): 462-4.
14. Ekouevi DK, Kariyiare BG, Coffie PA, et al. Feasibility and acceptability of rapid HIV screening in a labour ward in Togo. *Journal of the International AIDS Society* 2013; **15**(2).
15. Garrard N, Peck J, Ruf M, Lockyer S. Opt-out HIV testing pilot in termination of pregnancy services - 11-month service evaluation. *HIV Med* 2010; **11**: 69.

16. Gaydos CA, Solis M, Hsieh YH, Jett-Goheen M, Nour S, Rothman RE. Use of tablet-based kiosks in the emergency department to guide patient HIV self-testing with a point-of-care oral fluid test. *International Journal of STD & AIDS* 2013; **24**(9): 716-21.

17. Gennotte AF, Semaille P, Ellis C, et al. Feasibility and acceptability of HIV screening through the use of rapid tests by general practitioners in a Brussels area with a substantial African community. *HIV Medicine* 2013; **14**(SUPPL.3): 57-60.

18. Herbert R, Ashraf AN, Yates TA, et al. Nurse-delivered universal point-of-care testing for HIV in an open-access returning traveller clinic. *HIV Med* 2012; **13**(8): 499-504.

19. Hooshyar D, Suris AM, Czarnogorski M, Lepage JP, Bedimo R, North CS. Rapid HIV testing experience at Veterans Affairs North Texas Health Care System's Homeless Stand Downs. *AIDS Care - Psychological and Socio-Medical Aspects of AIDS/HIV* 2014; **26**(1): 95-9.

20. Jabbari H, Aghamollaie S, Esmaeeli Djavid G, et al. Frequency of HIV Infection among Sailors in South of Iran by Rapid HIV Test. *AIDS Res Treat* 2011; **2011**: 612475.

21. Jabbari H, Sharifi AH, SeyedAlinaghi S, et al. Assessing the prevalence of HIV among Afghan immigrants in Iran through rapid HIV testing in the field. *Acta Med Iran* 2011; **49**(7): 478-9.

22. Kania D, Fao P, Valéa D, et al. Low prevalence rate of indeterminate serological human immunodeficiency virus results among pregnant women from Burkina Faso, West Africa. *J Clin Microbiol* 2010; **48**(4): 1333-6.

23. Levin M, Mathema H, Stinson K, Jennings K. Acceptability, feasibility and impact of routine screening to detect undiagnosed HIV infection in 17 - 24-month-old children in the western sub-district of Cape Town. *S Afr Med J* 2012; **102**(4): 245-8.

24. Macgowan R, Margolis A, Richardson-Moore A, et al. Voluntary rapid human immunodeficiency virus (HIV) testing in jails. *Sex Transm Dis* 2009; **36**(2 Suppl): S9-13.

25. Manavi K, Williams G, Newton R. The uptake of HIV and syphilis testing in a nurse-delivered service during Gay Pride events. *International Journal of STD and AIDS* 2012; **23**(12): 887-9.

26. Martin EG, Salaru G, Paul SM, Cadoff EM. Use of a rapid HIV testing algorithm to improve linkage to care. *J Clin Virol* 2011; **52 Suppl 1**: S11-5.

27. Mathe MK, Rigo J, Sontag D, Gerard C. Prevalence of HIV infection among pregnant women. A study in rural Africa. [French] Prevalence de l'infection par le VIH chez les femmes enceintes. Etude en milieu rural africain. *Rev Epidemiol Sante Publique* 2008; **56**(6): 407-13.

28. Melo M, Varella I, Castro A, et al. HIV voluntary counseling and testing of couples during maternal labor and delivery: The TRIPAI couples study. *Sexually Transmitted Diseases* 2013; **40**(9): 704-9.

29. Melvin AJ, Alarcon J, Velasquez C, et al. Rapid HIV type 1 testing of women presenting in late pregnancy with unknown HIV status in Lima, Peru. *AIDS Res Hum Retroviruses* 2004; **20**(10): 1046-52.

30. Menacho I, Sequeira E, Muns M, et al. Comparison of two HIV testing strategies in primary care centres: Indicator-condition-guided testing vs. testing of those with non-indicator conditions. *HIV Medicine* 2013; **14**(SUPPL.3): 33-7.

31. Mkwanazi NB, Patel D, Newell ML, et al. Rapid testing may not improve uptake of HIV testing and same day results in a rural South African community: a cohort study of 12,000 women. *PLoS One* 2008; **3**(10): e3501.

32. Mullins TLK, Braverman PK, Dorn LD, Kollar LM, Kahn JA. Adolescent preferences for human immunodeficiency virus testing methods and impact of rapid tests on receipt of results. *Journal of Adolescent Health* 2010; **46**(2): 162-8.

33. Mungrue K, Sahadool S, Evans R, et al. Assessing the HIV rapid test in the fight against the HIV/AIDS epidemic in Trinidad. *HIV/AIDS - Research and Palliative Care* 2013; **5**: 191-8.

34. Ndondoki C, Brou H, Timite-Konan M, et al. Universal HIV Screening at Postnatal Points of Care: Which Public Health Approach for Early Infant Diagnosis in Cote d'Ivoire? *PLoS ONE* 2013; **8**(8).

35. Nelson AK, Caldas A, Sebastian JL, et al. Community-based rapid oral human immunodeficiency virus testing for tuberculosis patients in Lima, Peru. *Am J Trop Med Hyg* 2012; **87**(3): 399-406.

36. Newbould C, Monrose C, Dodge J, et al. Don't forget the children - Ongoing experience of a paediatric HIV unit using point-of-care tests in children born to HIV-positive parents - How far have we come? *HIV Med* 2010; **11**: 67-8.

37. Noble H, Wright G, Young E. HIV point of care testing in the emergency department. *HIV Med* 2012; **13**: 61.

38. Ouladlahsen A, Bensghir R, Karkouri M, et al. [Benefit of the rapid test determine HIV1/2 in the clinical diagnosis of HIV infection in Ibn Rochd hospital of Casablanca, Morocco]. *Rev Epidemiol Sante Publique* 2012; **60**(4): 333-8.

39. Pai NP, Barick R, Tulsky JP, et al. Impact of round-the-clock, rapid oral fluid HIV testing of women in labor in rural India. *PLoS Med* 2008; **5**(5): e92.

40. Parisi MR, Soldini L, Vidoni G, et al. Cross-sectional study of community serostatus to highlight undiagnosed HIV infections with oral fluid HIV-1/2 rapid test in non-conventional settings. *New Microbiologica* 2013; **36**(2): 121-32.

41. Ramachandran R, Chandrasekaran V, Muniyandi M, Jaggarajamma K, Bagchi A, Sahu S. Prevalence and Risk Factors of HIV Infection among Clients Attending ICTCs in Six Districts of Tamilnadu, South India. *AIDS Res Treat* 2011; **2011**: 650321.

42. Robbins CL, Zapata L, Kissin DM, et al. Multicity HIV seroprevalence in street youth, Ukraine. *Int J STD AIDS* 2010; **21**(7): 489-96.

43. Ruutel K, Ustina V, Parker RD. Piloting HIV rapid testing in community-based settings in Estonia. *Scand J Public Health* 2012; **40**(7): 629-33.

44. Sattin RW, Wilde JA, Freeman AE, Miller KM, Dias JK. Rapid HIV testing in a southeastern emergency department serving a semiurban-semirural adolescent and adult population. *Ann Emerg Med* 2011; **58**(1 Suppl 1): S60-4.

45. Scognamiglio P, Chiaradia G, Sciarrone MR, et al. Final results of an outreach program of HIV rapid testing among marginalized people living in Rome, Italy. *Infection* 2011; **39**: S24.

46. Stenstrom R, Grafstein E, Poureslami I, et al. Feasibility of and patient satisfaction withf HIV point-of-care testing in the emergency department. *Canadian Journal of Emergency Medicine* 2013; **15**: S30.

47. Tepper NK, Farr SL, Danner SP, et al. Rapid human immunodeficiency virus testing in obstetric outpatient settings: the MIRIAD study. *Am J Obstet Gynecol* 2009; **201**(1): 31 e1-6.

48. Theron GB, Shapiro DE, Van Dyke R, et al. Rapid intrapartum or postpartum HIV testing at a midwife obstetric unit and a district hospital in South Africa. *Int J Gynaecol Obstet* 2011; **113**(1): 44-9.

49. van Rooyen H, Barnabas RV, Baeten JM, et al. High HIV testing uptake and linkage to care in a novel program of home-based HIV counseling and testing with facilitated referral in KwaZulu-Natal, South Africa. *Journal of Acquired Immune Deficiency Syndromes: JAIDS* 2013; **64**(1): e1-8.

50. Veloso VG, Bastos FI, Portela MC, et al. HIV rapid testing as a key strategy for prevention of mother-to-child transmission in Brazil. *Rev Saude Publica* 2010; **44**(5): 803-11.

51. Viani RM, Araneta MRG, Spector SA. Parallel rapid HIV testing in pregnant women at Tijuana General Hospital, Baja California, Mexico. *AIDS Research and Human Retroviruses* 2013; **29**(3): 429-34.

52. White DA, Scribner AN, Schulden JD, Branson BM, Heffelfinger JD. Results of a rapid HIV screening and diagnostic testing program in an urban emergency department. *Ann Emerg Med* 2009; **54**(1): 56-64.

53. Young PW, Mahomed M, Horth RZ, Shiraishi RW, Jani IV. Routine data from prevention of mother-to-child transmission (PMTCT) HIV testing not yet ready for HIV surveillance in Mozambique: a retrospective analysis of matched test results. *BMC Infect Dis* 2013; **13**: 96.

54. Becker ML, Thompson LH, Pindera C, et al. Feasibility and success of HIV point-of-care testing in an emergency department in an urban Canadian setting. *Canadian Journal of Infectious Diseases and Medical Microbiology* 2013; **24**(1): 27-31.

55. Castel AD, Magnus M, Peterson J, et al. Implementing a novel citywide rapid HIV testing campaign in Washington, D.C.: findings and lessons learned. *Public Health Rep* 2012; **127**(4): 422-31.

56. Conners EE, Hagedorn HJ, Butler JN, et al. Evaluating the implementation of nurse-initiated HIV rapid testing in three Veterans Health Administration substance use disorder clinics. *International Journal of STD and AIDS* 2012; **23**(11): 799-805.

57. Guenter D, Greer J, Barbara A, Robinson G, Roberts J, Browne G. Rapid point-of-care HIV testing in community-based anonymous testing program: a valuable alternative to conventional testing. *AIDS Patient Care STDS* 2008; **22**(3): 195-204.

58. Nobrega I, Dantas P, Rocha P, et al. Syphilis and HIV-1 among parturient women in Salvador, Brazil: Low prevalence of syphilis and high rate of loss to follow-up in HIV-infected women. *Brazilian Journal of Infectious Diseases* 2013; **17**(2): 184-93.

59. Russell TV, Do AN, Setik E, et al. Sexual risk behaviors for HIV/AIDS in Chuuk State, Micronesia: the case for HIV prevention in vulnerable remote populations. *PLoS One* 2007; **2**(12): e1283.

60. Seewald R, Bruce RD, Elam R, et al. Effectiveness and feasibility study of routine HIV rapid testing in an urban methadone maintenance treatment program. *American Journal of Drug and Alcohol Abuse* 2013; **39**(4): 247-51.

61. Thomas R, Machouf N, Trottier B, et al. A new approach to encourage HIV testing in high-risk populations at the clinique l'actuel. *Sex Transm Infect* 2011; **87**: A201.

62. Keller S, Jones J, Erbelding E. Choice of Rapid HIV testing and entrance into care in Baltimore City sexually transmitted infections clinics. *AIDS Patient Care STDS* 2011; **25**(4): 237-43.

63. Marsh KA, Reynolds GL, Rogala BE, Fisher DG, Napper LE. Who chooses a rapid test for HIV in Los Angeles County, California? *Eval Health Prof* 2010; **33**(2): 177-96.

64. Benzaken A, Pinto VM, Carvalho CH, Peeling R. Increasing access to hiv and syphilis screening in remote areas using rapid tests. *Sex Transm Infect* 2011; **87**: A2.

65. Ganesan A, Thatchinamoorthy G, Saramini S. Feasibility of testing antibodies to HIV from filter paper using HIV rapid test kits. *J Int AIDS Soc* 2010; **13**.

66. Hoyos J, de la Fuente L, Fernandez S, et al. [Street outreach rapid HIV testing in university settings: a priority strategy?]. *Gac Sanit* 2012; **26**(2): 131-7.

67. Jerene D, Endale A, Lindtjorn B. Acceptability of HIV counselling and testing among tuberculosis patients in south Ethiopia. *BMC Int Health Hum Rights* 2007; **7**: 4.

68. Mikolasova G, Bonnach C, Monte CTW, et al. Relative low number of new hiv cases detected in rural district Bunda in Northwest Tanzania. *American Journal of Tropical Medicine and Hygiene* 2013; **1)**: 234.

69. Morin SF, Khumalo-Sakutukwa G, Charlebois ED, et al. Removing barriers to knowing HIV status: same-day mobile HIV testing in Zimbabwe. *J Acquir Immune Defic Syndr* 2006; **41**(2): 218-24.

70. Mwembo-Tambwe ANK, Kalenga MK, Donnen P, et al. HIV testing among women in delivery rooms in Lubumbashi, DR Congo: A catch-up strategy for prevention of mother-to-child transmission. [French] Depistage du VIH en salle de travail a Lubumbashi, Republique democratique du Congo. Une strategie de rattrapage dans le cadre de la prevention de la transmission de la mere a l'enfant. *Revue d'Epidemiologie et de Sante Publique* 2013; **61**(1): 21-7.

71. Portman M, Aung S, Brigstock-Barron O, et al. T'HIV'K goes North: Outcomes and experience of UK HIV Testing week in a northern UK city. *HIV Medicine* 2013; **14**: 19.

72. Qvist T, Cowan SA, Graugaard C, Helleberg M. High linkage to care in a community-based rapid HIV testing and counseling project among men who have sex with men in copenhagen. *Sexually Transmitted Diseases* 2014; **41**(3): 209-14.

73. Anaya HD, Hoang T, Golden JF, et al. Improving HIV screening and receipt of results by nurse-initiated streamlined counseling and rapid testing. *J Gen Intern Med* 2008; **23**(6): 800-7.

74. Beckwith CG, Liu T, Bazerman LB, et al. HIV risk behavior before and after HIV counseling and testing in jail: a pilot study. *J Acquir Immune Defic Syndr* 2010; **53**(4): 485-90.

75. Hernandez B, Mateo N, Walter J, Setterholm K. Targeted bedside emergency department hiv screening does not impact length of stay. *Academic Emergency Medicine* 2013; **1)**: S200.

76. Hoyos J, Fernandez-Balbuena S, de la Fuente L, et al. Never tested for HIV in Latin-American migrants and Spaniards: prevalence and perceived barriers. *J Int AIDS Soc* 2013; **16**: 18560.

77. Mayhood MK, Afwamba IA, Odhiambo CO, et al. Validation, performance under field conditions, and cost-effectiveness of Capillus HIV-1/HIV-2 and determine HIV-1/2 rapid human immunodeficiency virus antibody assays using sequential and parallel testing algorithms in Tanzania. *J Clin Microbiol* 2008; **46**(12): 3946-51.

78. Mehta SD, Hall J, Greenwald JL, Cranston K, Skolnik PR. Patient risks, outcomes, and costs of voluntary HIV testing at five testing sites within a medical center. *Public Health Rep* 2008; **123**(5): 608-17.

79. Metsch LR, Feaster DJ, Gooden L, et al. Implementing rapid HIV testing with or without risk-reduction counseling in drug treatment centers: results of a randomized trial. *Am J Public Health* 2012; **102**(6): 1160-7.

80. Puro V, Francisci D, Sighinolfi L, et al. Benefits of a rapid HIV test for evaluation of the source patient after occupational exposure of healthcare workers. *J Hosp Infect* 2004; **57**(2): 179-82.

81. Schulden JD, Song B, Barros A, et al. Rapid HIV testing in transgender communities by community-based organizations in three cities. *Public Health Rep* 2008; **123 Suppl 3**: 101-14.
